# Perioperative Management of Antirheumatic Medications in Adults with Rheumatoid Arthritis Undergoing Arthroplasty and Other Orthopedic Procedures: A Systematic Review

**DOI:** 10.3390/medicina62081498

**Published:** 2026-08-04

**Authors:** Ibrahim Al-Obaidi, Ibrahim Alabid, Afra Al-Dhaheri, Zain Al-Abdeen Mohammed Qassim, Mohamedanas Mohamedfaruk Patni, Mohamed El-Tanani, Syed Arman Rabbani, Aesha Shuaeeb, Radwan Abdulaziz Aloti, Wasim I. I. Alghoul

**Affiliations:** 1 Sheikh Tahnoon Bin Mohammed Medical City, PureHealth, Al Ain P.O. Box 15258, United Arab Emirates; ibrahimduraid13@gmail.com (I.A.-O.); aesha.salih2001@gmail.com (A.S.); 2 RAK College of Medical Sciences, RAK Medical and Health Sciences University, Ras Al Khaimah 11172, United Arab Emiratesradwan.21901025@rakmhsu.ac.ae (R.A.A.); wasim.21901111@rakmhsu.ac.ae (W.I.I.A.); 3 Rheumatology Department, Tawam/Sheikh Tahnoon Bin Mohammed Medical City Hospital, PureHealth, Al Ain P.O. Box 15258, United Arab Emirates; 4 Department of Community Medicine, RAK College of Medical Sciences, RAK Medical and Health Sciences University, Ras Al Khaimah 11172, United Arab Emirates; 5 RAK College of Pharmacy, RAK Medical and Health Science University, Ras Al Khaimah 11172, United Arab Emirates; eltanani@rakmhsu.ac.ae (M.E.-T.); arman@rakmhsu.ac.ae (S.A.R.)

**Keywords:** rheumatoid arthritis, perioperative management, disease-modifying antirheumatic drugs, biologics, JAK inhibitors, glucocorticoids, orthopedic surgery, arthroplasty, surgical site infection, delayed wound healing, flare

## Abstract

Perioperative management of antirheumatic medications in adults with rheumatoid arthritis (RA) undergoing orthopedic surgery requires balancing postoperative infection and impaired wound healing against disease flare, pain, and delayed rehabilitation. This systematic review aimed to evaluate how perioperative continuation, withholding, interruption timing, restart timing, dose modification, or exposure to antirheumatic medications relates to postoperative outcomes in adults with RA undergoing arthroplasty and non-arthroplasty orthopedic procedures. PubMed, Scopus, and the Cochrane Central Register of Controlled Trials were searched from database inception to 18 April 2026 for English-language full-text clinical studies. The primary outcomes were surgical site infection/periprosthetic joint infection, delayed wound healing, and postoperative RA flare. Secondary outcomes included reoperation, revision, readmission, mortality, venous thromboembolism, dislocation, and other procedure-specific complications. Original studies were classified as direct perioperative strategy evidence, medication-exposure evidence, or supportive clinical-context evidence. Forty-three original clinical studies were included, together with six non-original contextual sources. The evidence was predominantly observational, clinically heterogeneous, and of very low certainty. Methotrexate continuation was not associated with an evident increase in early postoperative complications, whereas direct evidence for other conventional synthetic agents was limited. Longer interruption of biologic or targeted synthetic therapy did not consistently reduce infection or wound-healing complications, while several studies associated treatment interruption with postoperative flare. Chronic baseline glucocorticoid exposure was associated with adverse outcomes but was clinically distinct from short-term perioperative administration; stress-dose supplementation was not directly evaluated. Medication decisions should therefore be individualized according to drug class, disease control, glucocorticoid burden, surgical procedure, and patient risk profile.

## 1. Introduction

Rheumatoid arthritis (RA) is a chronic systemic inflammatory disease characterized by persistent synovitis, progressive structural joint damage, pain, disability, and impaired quality of life. Despite major advances in disease-modifying treatment, RA continues to impose a substantial global burden. An estimated 17.6 million people were living with RA worldwide in 2020, and this number is projected to increase to approximately 31.7 million by 2050, largely because of population growth and aging [1]. Contemporary treatment strategies emphasize early diagnosis, treat-to-target monitoring, and timely escalation of conventional synthetic, biologic, or targeted synthetic disease-modifying antirheumatic drugs (DMARDs) to suppress inflammation, maintain function, and prevent irreversible structural damage [2].

Orthopedic manifestations of RA result from the combined effects of persistent synovitis, cartilage loss, marginal bone erosion, periarticular osteopenia, ligamentous attenuation, tendon attrition or rupture, joint instability, and progressive deformity. In the hand and wrist, these pathological processes may produce metacarpophalangeal subluxation, ulnar drift, tendon imbalance, carpal collapse, and loss of grip function. Foot and ankle involvement may include metatarsophalangeal subluxation, hallux valgus, hindfoot deformity, ankle destruction, and compromised soft-tissue integrity. Large-joint disease may progress to end-stage hip or knee destruction, while cervical involvement may cause atlantoaxial or subaxial instability. These characteristic abnormalities may complicate patient positioning, airway and anesthetic management, fixation, wound closure, implant stability, postoperative mobilization, and rehabilitation [3,4,5,6,7,8,9].

Modern pharmacological treatment has reduced the overall requirement for orthopedic intervention, but it has not eliminated surgery from RA care. Orthopedic procedures remain necessary for patients with persistent pain, irreversible structural destruction, mechanical failure, instability, tendon dysfunction, neurological compromise, or severe deformity that cannot be controlled adequately by medical therapy. The distribution of surgical procedures has also changed rather than disappeared. A 20-year observational cohort reported an approximately 50% reduction in the overall frequency of RA-related orthopedic surgery, while foot and ankle procedures increased by approximately 67% during the same period [10]. Contemporary RA surgery, therefore, extends beyond total hip arthroplasty (THA) and total knee arthroplasty (TKA) to include hand and wrist reconstruction, forefoot correction, total ankle arthroplasty, cervical spine surgery, revision surgery, and other arthroplasty and non-arthroplasty procedures [3,4,5,6,7,8].

Perioperative decision making is further complicated by the diversity of antirheumatic therapies. Conventional synthetic DMARDs (csDMARDs) include methotrexate, leflunomide, hydroxychloroquine, and sulfasalazine. Biologic DMARDs (bDMARDs) include tumor necrosis factor inhibitors and non-tumor necrosis factor agents that target interleukin-6 signaling, T-cell costimulation, B cells, and other inflammatory pathways. Targeted synthetic DMARDs (tsDMARDs), principally Janus kinase inhibitors, have distinct pharmacokinetic and immunomodulatory profiles and are increasingly used in patients with an inadequate response to other agents [2]. Glucocorticoid exposure also requires distinction between chronic baseline therapy, short-term perioperative administration, and perioperative stress-dose supplementation. These medication classes differ in their mechanisms, dosing intervals, duration of action, and immunosuppressive effects and, therefore, should not be assumed to carry identical perioperative risks or require identical interruption strategies [2,11,12].

Continuing immunomodulatory treatment throughout the perioperative period may raise concern for surgical site infection (SSI), periprosthetic joint infection (PJI), impaired wound healing, or other postoperative complications. Conversely, withholding treatment may precipitate disease flare, worsen pain and stiffness, restrict early mobilization, delay rehabilitation, and compromise functional recovery. The balance between these competing risks may vary according to the specific medication, baseline disease activity, chronic glucocorticoid burden, previous infection or revision surgery, anatomical site, operative complexity, implant dependence, local soft-tissue condition, and consequences of postoperative immobility [4,8,13,14].

Current perioperative guidelines have helped standardize practice but are weighted primarily toward patients undergoing elective THA and TKA [11,12]. Their direct applicability to hand, foot, ankle, spine, revision, and other non-arthroplasty procedures remains uncertain because these operations differ in wound burden, soft-tissue quality, implant dependence, biomechanical requirements, and rehabilitation priorities. Previous evidence syntheses have also frequently concentrated on particular medication classes, infection outcomes, selected procedures, or mixed inflammatory-arthritis populations [8,13,14]. Consequently, important uncertainty remains regarding how arthroplasty-derived recommendations should be applied across the broader spectrum of RA-related orthopedic surgery.

The clinical significance of the present review lies in its RA-specific evaluation of perioperative antirheumatic medication management across both arthroplasty and non-arthroplasty orthopedic procedures. Unlike syntheses restricted primarily to infection or large-joint arthroplasty, this review evaluates SSI/PJI, delayed wound healing, postoperative RA flare, and other clinically relevant complications across csDMARDs, bDMARDs, tsDMARDs, and glucocorticoids. It also distinguishes direct perioperative strategy evidence from medication-exposure associations and supportive clinical-context evidence. This evidence hierarchy is intended to clarify which conclusions are supported directly by comparisons of continuation, withholding, or interruption timing and which remain dependent on observational associations, guideline extrapolation, or multidisciplinary clinical interpretation.

This systematic review aimed to evaluate how perioperative continuation, withholding, interruption timing, restart timing, dose modification, and exposure to antirheumatic medications relate to postoperative outcomes in adults with RA undergoing arthroplasty and non-arthroplasty orthopedic procedures. The primary outcomes were SSI/PJI, delayed wound healing, and postoperative RA flare. A secondary objective was to characterize disease-, patient-, procedure-, and postoperative interpretive factors that may modify or confound medication–outcome associations.

## 2. Materials and Methods

### 2.1. Study Design and Registration

This systematic review was reported in accordance with the Preferred Reporting Items for Systematic Reviews and Meta-Analyses (PRISMA) 2020 statement [15]. Its primary purpose was to evaluate how perioperative continuation, withholding, interruption timing, restart timing, dose modification, and exposure to antirheumatic medications relate to postoperative outcomes in adults with RA undergoing arthroplasty and non-arthroplasty orthopedic procedures. The primary outcomes were surgical site infection/periprosthetic joint infection, delayed wound healing, and postoperative RA flare. A secondary objective was to characterize disease-, patient-, procedure-, and postoperative interpretive factors that may modify or confound medication–outcome associations.

To avoid conflating direct treatment evidence with broader clinical associations, original studies were classified according to their evidentiary role as direct perioperative strategy evidence, medication-exposure evidence, or supportive clinical-context evidence. Supportive studies informed interpretation of surgical selection, disease activity, procedure-related risk, or postoperative findings but were not used to determine whether a medication should be continued or withheld.

The protocol was registered in the International Prospective Register of Systematic Reviews (PROSPERO) on 18 April 2026 under registration number CRD420261372707. No changes were made to the registered population, information sources, primary outcomes, or principal review question. During manuscript revision, the original studies were classified more explicitly according to evidence role to distinguish direct medication evidence from supportive clinical-context evidence.

### 2.2. PICOS Statement and Evidence Framework

The primary review question was structured according to the PICOS framework.

Population: Adults aged 18 years or older with RA undergoing arthroplasty or non-arthroplasty orthopedic procedures.

Intervention/Exposure: Perioperative continuation, discontinuation, temporary withholding, interruption duration, restart timing, dose modification, or exposure to conventional synthetic, biologic, or targeted synthetic disease-modifying antirheumatic drugs or glucocorticoids.

Comparator: Continuation versus withholding, shorter versus longer interruption, medication-modified versus unmodified care, medication-exposed versus unexposed groups, different medication classes, or glucocorticoid dose strata.

Outcomes: The primary clinical outcomes were surgical site infection/periprosthetic joint infection, delayed wound healing, and postoperative RA flare. Secondary clinical outcomes included reoperation, revision, readmission, mortality, venous thromboembolism, dislocation, mechanical complications, and other procedure-specific postoperative complications.

Study designs: Randomized and non-randomized original clinical studies, including prospective and retrospective cohorts, case–control studies, registry studies, administrative database studies, comparative observational studies, and non-comparative studies describing a defined perioperative medication strategy.

A supplementary evidence framework was used for original studies that did not directly compare medication strategies but provided clinically relevant information concerning the interpretation of perioperative evidence. These included studies of disease activity, surgical utilization, anatomical or procedure-specific risk, previous infection or revision, operative complexity, and postoperative inflammatory-marker patterns. Such studies were classified as supportive clinical-context evidence and were not used to infer the causal effect of medication continuation or withholding. Guidelines, protocols, systematic reviews, meta-analyses, and focused reviews were retained only as non-original contextual sources.

### 2.3. Eligibility Criteria

Studies were eligible under either the primary medication-evidence framework or the supportive clinical-context framework.

Primary medication-evidence inclusion criteria:

• Population: Adults aged 18 years or older with RA undergoing arthroplasty or non-arthroplasty orthopedic procedures.
• Medication intervention or exposure: Perioperative continuation, discontinuation, temporary withholding, interruption timing, restart timing, dose modification, short-term perioperative administration, chronic baseline exposure, or medication-exposed versus unexposed comparisons involving csDMARDs, bDMARDs, tsDMARDs, JAK inhibitors, or glucocorticoids.
• Clinical outcomes: At least one postoperative clinical outcome, including SSI, PJI, DWH, postoperative RA flare, reoperation, revision, readmission, mortality, VTE, dislocation, mechanical complications, or another procedure-specific complication.
• Postoperative assessment period: Eligible studies were required to report at least one clinical outcome assessed after the index orthopedic procedure. No prespecified minimum or maximum postoperative follow-up duration was imposed because the outcomes of interest were evaluated across heterogeneous but clinically relevant postoperative intervals. The time from surgery to outcome assessment and the total follow-up duration were recorded whenever reported.
• Study designs: Randomized controlled trials and non-randomized original clinical studies, including prospective or retrospective cohorts, case–control studies, registry studies, administrative database studies, and comparative observational studies. Non-comparative studies were eligible when they described a defined perioperative medication strategy and reported at least one clinically relevant postoperative outcome.

Supportive clinical-context inclusion criteria:

Original clinical studies involving adults aged 18 years or older with RA undergoing orthopedic surgery were also eligible when they directly informed interpretation of the primary medication evidence by evaluating one or more of the following:

• disease activity or inflammatory burden;
• demographic or disease-related determinants of orthopedic surgery utilization;
• previous infection, primary or revision surgery, or surgical complexity;
• procedure type, anatomical site, or operative duration;
• postoperative inflammatory-marker kinetics or other clinically relevant interpretive measures;
• patient-level factors capable of confounding medication–outcome associations.

These studies were classified separately and were not used to infer that medication continuation, withholding, or timing modification caused a postoperative outcome. Time since RA diagnosis was not used as an eligibility criterion or prespecified subgroup variable because it was inconsistently reported across the available literature. Consequently, no formal analysis according to RA duration was undertaken.

Exclusion criteria:

• Narrative reviews, systematic reviews, meta-analyses, scoping reviews, editorials, expert opinions, clinical guidelines, study protocols, conference abstracts without sufficient extractable data, and case reports.
• Non-human studies.
• Non-orthopedic surgical studies.
• Studies with no RA population or no separable RA-relevant evidence, except mixed inflammatory-arthritis cohorts that directly evaluated perioperative antirheumatic medication exposure and were interpreted cautiously as indirect supportive evidence.
• Studies focused solely on long-term implant survivorship without perioperative medication relevance or clinically relevant RA orthopedic context.
• Studies not published as full-text articles in English.

Non-original records excluded from the systematic clinical evidence base because of study design were retained as contextual sources only when they directly informed guideline interpretation, previous evidence synthesis, or research gaps.

### 2.4. Information Sources and Search Strategy

A comprehensive literature search was conducted in PubMed, Scopus, and the Cochrane Central Register of Controlled Trials (CENTRAL) from database inception to 18 April 2026. Searches were limited to English-language full-text publications. The search strategy combined controlled-vocabulary and free-text terms related to rheumatoid arthritis, orthopedic surgery, perioperative management, and antirheumatic medication exposure. Core search terms included “Arthritis, Rheumatoid”, “rheumatoid arthritis”, orthopedic*, orthopaedic*, surgery, arthroplasty, hip arthroplasty, knee arthroplasty, hand surgery, wrist surgery, foot surgery, ankle surgery, spine surgery, perioperative, preoperative, postoperative, DMARD*, biologic*, targeted synthetic, JAK inhibitor*, glucocorticoid*, steroid*, methotrexate, leflunomide, infliximab, abatacept, tocilizumab, rituximab, adalimumab, etanercept, and ozoralizumab. Search syntax was adapted to the indexing system of each database. Additional potentially relevant studies were identified through manual reference list screening and forward citation tracking. Original studies were retained within the review when they met either the primary medication-evidence criteria or the supportive clinical-context criteria. Guideline papers, protocols, systematic reviews, meta-analyses, and focused reviews were retained only for contextual interpretation and were not included among the 43 original clinical studies. Before formal title and abstract screening, records were processed using prespecified database and metadata-based eligibility rules. Records were removed when database metadata identified a non-English publication, an ineligible publication type, a non-human or laboratory-only study, or a record clearly outside the prespecified rheumatoid arthritis–orthopedic surgery scope. These exclusions were completed before reviewer-based title and abstract screening and accounted for 479 records. The pre-screening exclusion categories and the complete electronic search strategies are reported in Supplementary S1. The full electronic search strategies for PubMed, Scopus, and CENTRAL, including all limits and filters applied, are provided in Supplementary S1.

### 2.5. Study Selection

All records retrieved from PubMed, Scopus, and CENTRAL were collated before formal screening. After removal of 211 duplicate records, 479 records were removed before title and abstract screening using prespecified database metadata, indexing terms, publication-type filters, and keyword-based scope rules. These records comprised non-English-language publications, ineligible publication types, non-human or laboratory-only studies, and records clearly outside the predefined rheumatoid arthritis–orthopedic surgery scope. The remaining 106 records underwent formal title and abstract screening, of which 57 were excluded because they did not meet the population, surgical setting, medication relevance, evidence-context, or study-design criteria.

Full texts were sought for 49 reports, all of which were successfully retrieved and assessed for eligibility. During full-text assessment, original clinical studies were classified according to their evidentiary role. Direct perioperative strategy studies evaluated medication continuation, withholding, interruption duration, restart timing, short-term perioperative administration, or medication modification. Medication-exposure studies compared medication-exposed and unexposed groups, different medication classes, or dose strata without necessarily testing a specific perioperative timing strategy. Supportive clinical-context studies evaluated disease-, patient-, procedure-, or postoperative interpretive factors relevant to surgical selection, perioperative risk, confounding, or postoperative assessment. All three categories were retained among the 43 original clinical studies; however, supportive clinical-context studies were not used as direct evidence that a medication should be continued or withheld.

Six non-original reports that did not meet the study-design eligibility criteria—one study protocol, two clinical guidelines, two systematic review or meta-analytic sources, and one focused review—were retained only for contextual interpretation and were not included in the primary medication-effect synthesis, risk-of-bias conclusions, or GRADE assessment.

**Figure 1:**
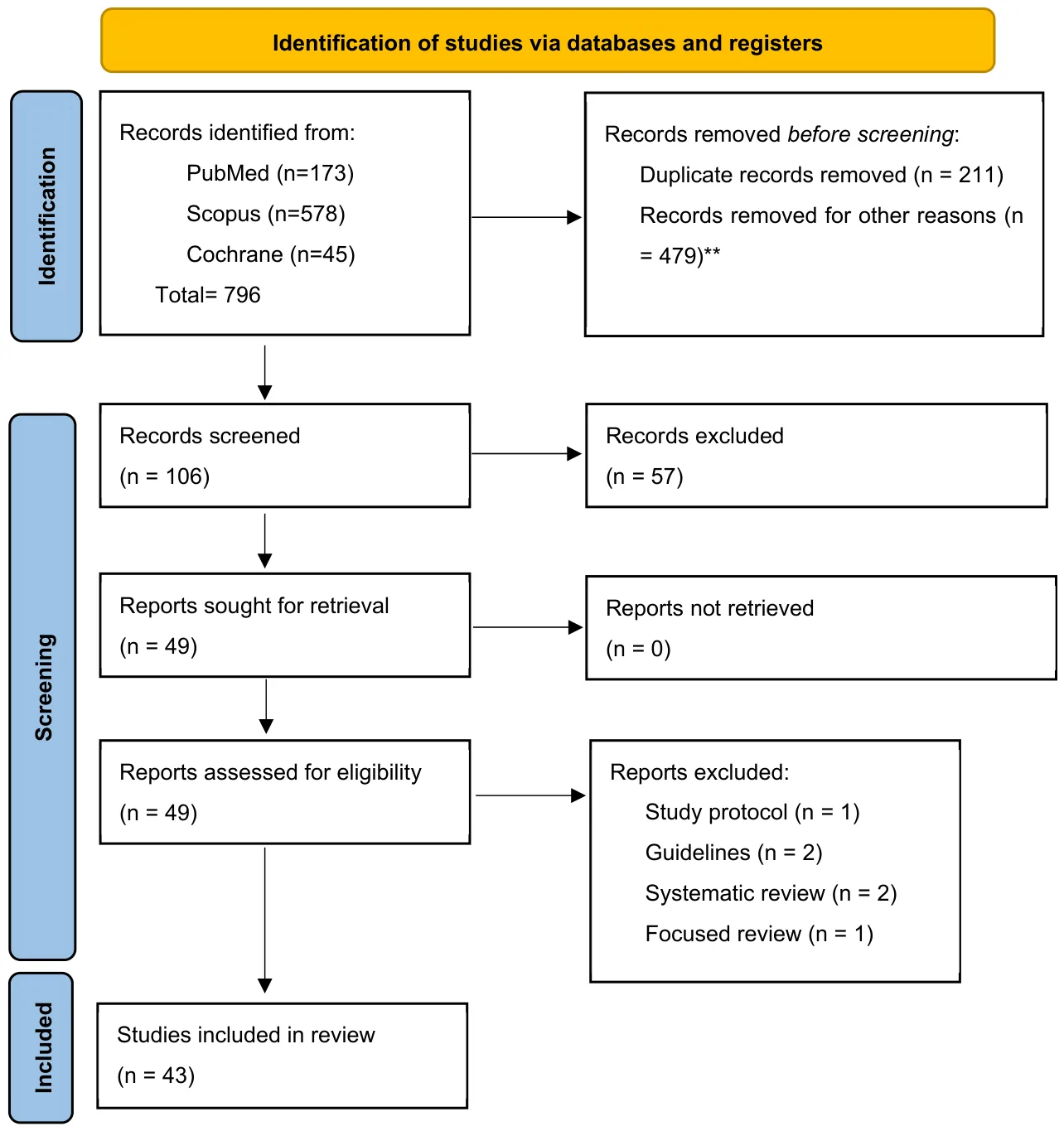
PRISMA flow diagram depicting the study selection process. ** Records were removed before formal screening when database metadata, indexing terms, or prespecified filters identified them as non-English-language publications, ineligible publication types, non-human or laboratory-only studies, or records clearly outside the predefined rheumatoid arthritis–orthopedic surgery scope.

Titles and abstracts and full-text reports were screened by one reviewer, with all decisions checked by at least one additional reviewer. Any uncertainties or disagreements were resolved through discussion. Where multiple reports appeared to arise from overlapping cohorts or related clinical settings, each report was evaluated according to its study period, population, surgical setting, exposure, and outcomes to avoid duplicate inclusion of the same outcome dataset. No machine-learning or automated eligibility-classification tools were used for title and abstract screening or full-text eligibility decisions. Database limits, metadata-based filters, and prespecified keyword rules were applied only before formal reviewer-based screening, as described above and in Supplementary S1. The complete study-selection process is presented in Figure 1.

### 2.6. Data Extraction

Data were extracted using a standardized data collection form by one reviewer and checked by at least one additional reviewer. The following variables were recorded:

• study characteristics: author, year, country, study design, and sample size;
• clinical setting: orthopedic procedure type and surgical category;
• perioperative exposure and postoperative timing: medication class, specific drug, interruption or continuation strategy, comparator group, time from surgery to each outcome assessment, and total follow-up duration;
• outcomes: SSI/PJI, DWH, postoperative flare, reoperation, revision, readmission, mortality, VTE, dislocation, and other procedure-specific complications;
• quantitative data: event counts, proportions, odds ratios, hazard ratios, risk ratios, confidence intervals, and *p* values where available.

Additional variables were extracted when reported, including disease activity, glucocorticoid burden, comorbidities, operative time, revision status, and study-specific outcome definitions. Individual participant data were not sought. Missing or unclear information was recorded as not reported; no assumptions or imputations were made. No automation tools were used during data extraction.

For each outcome domain, all compatible postoperative results reported by the study authors were sought when available, including different definitions, time points, and adjusted or unadjusted estimates. When multiple estimates were reported, adjusted measures were prioritized over crude data.

Each original study was additionally assigned an evidence-role classification: direct perioperative strategy evidence, medication-exposure evidence, or supportive clinical-context evidence. For supportive studies, the specific contextual role was recorded, such as disease activity, surgical utilization, anatomical risk, operative complexity, previous infection or revision, or postoperative biomarker interpretation.

### 2.7. Risk of Bias Assessment

Risk of bias was assessed using the Cochrane Risk of Bias 2 (RoB 2) tool for randomized studies and the Newcastle–Ottawa Scale for non-randomized cohort and case–control studies. Assessment was performed by two reviewers and checked by at least one additional reviewer.

Particular attention was given to selection bias, confounding by indication, baseline imbalance in disease severity, heterogeneity in perioperative medication-hold intervals, inconsistency in outcome definitions, variable follow-up duration, and incomplete adjustment for major clinical confounders such as glucocorticoid exposure, revision surgery, disease activity, and prior infection history.

Risk of bias due to missing results was considered through assessment of selective outcome reporting, incomplete outcome reporting, and missing key data in included studies. No additional information was sought from study investigators. No automation tools were used for risk-of-bias assessment.

Risk-of-bias assessment was performed for all 43 original studies, including supportive clinical-context studies. Inclusion in the risk-of-bias visualization indicates that a study contributed original clinical data; it does not imply that the study provided direct evidence regarding medication continuation or withholding.

### 2.8. Data Synthesis and Statistical Analysis

Because substantial clinical and methodological heterogeneity was expected across medication classes, orthopedic procedure types, comparator strategies, perioperative interruption intervals, and outcome definitions, the synthesis was planned as a structured narrative synthesis rather than a quantitative meta-analysis. The review retained 43 original clinical studies, which were classified according to their evidentiary role as direct perioperative strategy evidence, medication-exposure evidence, or supportive clinical-context evidence.

Direct perioperative strategy studies evaluated medication continuation versus withholding, shorter versus longer interruption, timing of the last preoperative dose, timing of postoperative restart, short-term perioperative administration, or medication modification. Medication-exposure studies compared medication-exposed and unexposed groups, different medication classes, or medication-dose strata without necessarily testing a specific perioperative timing strategy. Supportive clinical-context studies evaluated disease-, patient-, procedure-, or postoperative interpretive factors that could modify, confound, or assist the interpretation of associations between medication exposure and postoperative outcomes.

Medication-management conclusions were based principally on direct perioperative strategy evidence and, with greater caution, medication-exposure evidence. Supportive clinical-context studies were used to explain clinical heterogeneity, potential confounding, postoperative interpretation, and the generalizability of the direct evidence, but were not interpreted as evidence that medication continuation, withholding, or modification caused a particular postoperative outcome. The six non-original contextual sources were used only to frame existing recommendations, compare the findings with previous evidence syntheses, and identify research gaps.

Studies were grouped first according to the primary outcome domains:

• surgical site infection/periprosthetic joint infection;
• delayed wound healing; and
• postoperative rheumatoid arthritis flare.

Studies were subsequently synthesized according to:

• secondary postoperative outcomes;
• medication class;
• orthopedic procedure type;
• perioperative continuation versus withholding strategy; and
• disease-, patient-, and procedure-related factors that might modify or confound postoperative risk, including disease activity, chronic baseline glucocorticoid burden, previous infection, revision status, anatomical site, and operative complexity.

Additional subgroup interpretation was performed for:

• conventional synthetic disease-modifying antirheumatic drugs versus biologic disease-modifying antirheumatic drugs versus targeted synthetic agents;
• arthroplasty versus non-arthroplasty procedures;
• hip and knee arthroplasty versus foot and ankle, hand and wrist, and spine surgery;
• continuation versus discontinuation or shorter versus longer interruption strategies; and
• studies emphasizing infection outcomes versus wound-healing outcomes versus flare outcomes.

Within each outcome domain, direct perioperative strategy evidence was considered first, followed by medication-exposure evidence and supportive clinical-context evidence. This approach was used to prevent indirect risk-factor or interpretive studies from being presented as equivalent to comparative medication-management studies.

Where available, adjusted effect estimates and clinically relevant event frequencies were prioritized over crude data. The principal effect measures extracted for infection, wound-healing, flare, and secondary complication outcomes included odds ratios, hazard ratios, risk ratios, proportions, and raw event counts, depending on the design and reporting of each study. When both adjusted and unadjusted estimates were available, adjusted estimates were prioritized. A meta-analysis was not undertaken because sufficient clinical and methodological homogeneity was not identified for a specific medication comparison, surgical setting, outcome definition, and follow-up period.

The certainty of evidence for each primary and key secondary outcome was assessed using the Grading of Recommendations Assessment, Development and Evaluation (GRADE) approach. Because the contributing evidence was predominantly observational, certainty generally began at low and was downgraded, when appropriate, for risk of bias, inconsistency, indirectness, imprecision, and suspected publication or reporting bias. Randomized evidence began at high certainty but was downgraded when limitations were identified. No rating up was applied because large effects were not sufficiently consistent across procedures or medication classes and residual confounding remained likely.

Medication-effect certainty judgments were based on studies contributing direct perioperative strategy or medication-exposure evidence to the relevant clinical outcome. Supportive clinical-context studies were retained in the broader narrative synthesis but were not used to increase the certainty of causal conclusions concerning medication continuation or withholding. In particular, studies addressing orthopedic surgery utilization or postoperative inflammatory-marker kinetics did not contribute to medication-effect GRADE ratings.

**Figure 2:**
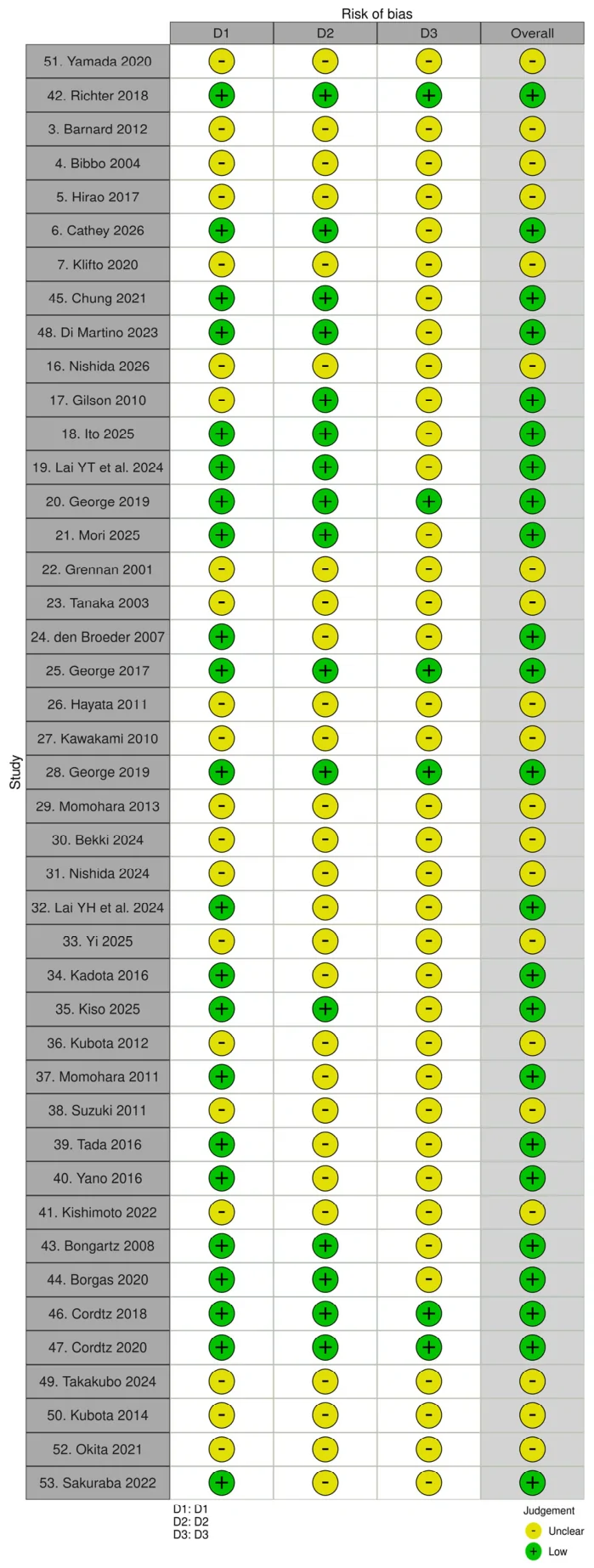
Adapted traffic-light summary of methodological quality and risk-of-bias judgments for the 43 original clinical studies included in the review. D1, selection; D2, comparability; D3, outcome/exposure; Overall, overall methodological judgment. Non-randomized cohort and case–control studies were assessed using the Newcastle–Ottawa Scale, whereas the randomized methotrexate trial was assessed using the Cochrane Risk of Bias 2 tool and harmonized into the same categories for visualization. Green (+) indicates low risk/good methodological quality, and yellow (−) indicates unclear risk/fair methodological quality. Inclusion in the figure indicates that a study contributed original clinical data and should not be interpreted as indicating directness of evidence for medication continuation or withholding. The six non-original contextual sources were excluded. Studies displayed: [3,4,5,6,7,16,17,18,19,20,21,22,23,24,25,26,27,28,29,30,31,32,33,34,35,36,37,38,39,40,41,42,43,44,45,46,47,48,49,50,51,52,53].

For non-randomized studies, overall methodological quality judgments were visualized using an adapted traffic-light plot (Figure 2). All 43 original clinical studies were included in the risk-of-bias assessment; however, inclusion in the risk-of-bias visualization indicated contribution of original clinical data and did not imply that every study directly evaluated a perioperative medication-management strategy.

### 2.9. Ethics and Data Availability

This study used published data only and did not involve direct human participant recruitment; therefore, ethical approval was not required. All data used in the review were derived from published articles included in the final evidence synthesis.

## 3. Results

### 3.1. Study Selection

The complete study-selection pathway, including duplicate removal, pre-screening exclusions, title and abstract screening, full-text assessment, and reasons for exclusion, is presented in Figure 1. Forty-three original clinical studies met the eligibility criteria, while six non-original sources were retained solely for contextual interpretation.

### 3.2. Study Characteristics

Detailed characteristics of the included studies are summarized in Table 1. The 43 original studies differed substantially in their directness for the review question. Direct strategy studies explicitly evaluated continuation, withholding, interruption duration, restart timing, short-term perioperative administration, or medication modification. Medication-exposure studies compared exposed and unexposed patients, medication classes, or dose strata without necessarily testing a modifiable perioperative timing strategy. Supportive clinical-context studies examined disease activity, surgical utilization, procedure-specific risk, previous infection or revision, operative complexity, or postoperative interpretation. This classification was maintained throughout the synthesis so that indirect contextual evidence was not used to support causal medication recommendations. The included evidence was dominated by observational designs, especially retrospective single-center, multicenter, registry-based, and administrative database studies. Study size varied markedly, from 9 patients undergoing 12 procedures in the ozoralizumab case series [16] and 20 infected arthroplasty cases with 40 matched controls in the French TNF-blocker case–control study [17], to 1953 patients in a contemporary multicenter bDMARD/tsDMARD cohort [18], 337 RA THAs in a disease-activity study [19], 10,923 arthroplasties in a U.S. biologic/glucocorticoid arthroplasty cohort [20], and 3951 RA THAs in a nationwide Japanese claims analysis [21].

The surgical spectrum was broad and clinically heterogeneous. Arthroplasty-focused studies included THA, TKA, TJA, and TAA cohorts, whereas non-arthroplasty and mixed orthopedic studies examined forefoot reconstruction, elective foot and ankle surgery, hand and upper-extremity surgery, cervical spine surgery, and mixed orthopedic cohorts spanning joint replacement, foot, hand, elbow, spine, and trauma-related procedures. This breadth strengthened clinical relevance but increased heterogeneity in infection risk, wound burden, flare risk, and outcome ascertainment.

The medication exposures of interest also varied substantially. Some studies isolated conventional agents, such as methotrexate continuation [22] or leflunomide continuation versus interruption [23], while others focused on anti-TNF therapy [4,17,24,25,26,27], non-TNF biologics such as abatacept [28] and tocilizumab [29], JAK inhibitors [30,31], perioperative glucocorticoid strategies [20,32,33], or very recent agents such as ozoralizumab [16]. Mixed biologic-era cohorts were common, especially in Japanese orthopedic series, where procedures performed under biologic treatment were compared with nonbiologic or mixed antirheumatic regimens [34,35,36,37,38,39].

Outcome definitions were inconsistent across the literature. Arthroplasty database studies frequently used hospitalized infection within 30 days and prosthetic joint infection within 1 year [20], whereas other cohorts used in-hospital endpoints only [21], 90-day postoperative surveillance [31], or 1-year follow-up for elective orthopedic procedures [39]. Delayed wound healing definitions were also variable: Yano et al. defined it as wounds not healed by >2 weeks after surgery [40], Tada et al. used physician-judged delayed healing after elective orthopedic procedures [39], Yi et al. used both delayed wound healing >4 weeks and serial ASEPSIS scoring after total ankle arthroplasty [33], and Nishida et al. defined delayed wound healing as stitch removal after >2 weeks or re-suturing in the ozoralizumab series [16]. Interpretation of postoperative inflammatory markers was also heterogeneous; in a biologic-era RA TKA cohort of 214 cases, serum CRP peaked on postoperative days 3–4 and returned near baseline by day 14, while BMI ≥ 25 and glucocorticoid use independently influenced CRP change, indicating that postoperative CRP kinetics may reflect host and treatment factors as well as complications [41]. These differences limited direct quantitative comparison across studies.

Two included original studies had a specifically supportive rather than medication-comparative role. Richter et al. evaluated demographic, disease-related, and temporal factors associated with orthopedic surgery among patients with RA [42]. This study informed the clinical context in which RA patients are selected for orthopedic surgery but did not evaluate a perioperative medication strategy. Kishimoto et al. evaluated postoperative CRP kinetics after TKA and identified BMI and glucocorticoid exposure as factors influencing postoperative inflammatory-marker changes [41]. This study informed interpretation of postoperative laboratory findings but did not establish the clinical safety or effectiveness of a medication continuation or withholding strategy. Neither study was used to formulate medication-timing recommendations or medication-effect GRADE conclusions. The evidence-role assignments for each original study are presented in Table 1 and Table S1.

**Table 1:** Detailed characteristics of included studies. (**A**). Arthroplasty-focused studies. (**B**). Non-arthroplasty and mixed orthopedic studies.

(A)								
Author & Year	Surgery Type	Study Design	Population Studied	N (Patients/Procedures)	Perioperative Medication Strategy/Exposure	Comparator	Primary Outcomes	Evidence Role
Hirao 2017 [5]	TAA	Retrospective study	Japanese RA patients undergoing TAA	50 ankles	Biologic-era TAA outcomes	Internal cohort comparisons	Implant survival, complications	S
Gilson 2010 [17]	Total joint arthroplasty	Case–control study	RA patients receiving TNF blockers undergoing TJA	20 infected cases/40 matched controls	TNF-blocker exposure and glucocorticoid burden	Infected vs. non-infected matched arthroplasty patients	Risk factors for TJA infection	E
Lai YT et al. 2024 [19]	THA	Retrospective cohort study	RA patients undergoing primary THA	337 patients	Disease activity at admission/follow-up	SDAI strata within RA cohort	Complications, infection, dislocation	S
George 2019 [20]	Arthroplasty	Cohort study	RA patients undergoing arthroplasty	10,923 procedures	Biologic exposure and chronic glucocorticoid burden	Different biologic agents; steroid dose strata	Hospitalized infection, 1-year PJI, readmission	E
Mori 2025 [21]	THA	Nationwide medical claims database study	RA vs. OA patients undergoing THA	3951 RA THAs after matching	Disease cohort comparison	RA vs. OA	Dislocation, reoperation, in-hospital complications	S
Tanaka 2003 [23]	Joint arthroplasty	Retrospective comparative study	RA patients undergoing arthroplasty	82 patients/161 arthroplasties	Continuous leflunomide treatment	Leflunomide vs. no leflunomide/alternative therapy	Infectious complications	D
George 2017 [25]	Elective hip/knee arthroplasty	Retrospective cohort	RA patients receiving infliximab undergoing arthroplasty	4288 surgeries in infliximab-treated immune-mediated disease cohort	Timing of infliximab interruption before surgery	Shorter vs. longer interruption	30-day serious infection, 1-year PJI	D
George 2019 [28]	Elective arthroplasty	Retrospective cohort	RA patients receiving abatacept undergoing arthroplasty	1780 patients/1939 surgeries	Timing of abatacept interruption before surgery	Shorter vs. longer interruption	Postoperative infection and other outcomes	D
Lai YH et al. 2024 [32]	Total joint arthroplasty	Retrospective cohort	RA patients undergoing TJA	849 patients	Short-term perioperative glucocorticoids	Glucocorticoid vs. no short-term glucocorticoid	Early complications, readmission, analgesic outcomes	D
Yi 2025 [33]	TAA	Retrospective comparative study	Severe RA patients undergoing TAA	34 patients/34 ankles	Perioperative medication modification	No medication modification	Early complications, DWH	D
Momohara 2011 [37]	THA/TKA	Retrospective cohort	RA patients undergoing THA/TKA	420 arthroplasties	Biologic and nonbiologic DMARD exposure	Nonbiologic vs. biologic exposure	PJI, acute SSI	E
Kishimoto 2022 [41]	TKA	Retrospective cohort	RA patients undergoing TKA in the biologic era	214 cases	Biologic-era perioperative inflammatory-marker profile	BMI and glucocorticoid strata	Postoperative CRP kinetics	S
Bongartz 2008 [43]	THA/TKA	Cohort study	RA patients undergoing total hip or knee replacement	657 arthroplasties	RA arthroplasty cohort; medication and clinical risk-factor assessment	Within-cohort risk-factor comparisons	PJI, risk factors	S
Borgas 2020 [44]	Elective THA/TKA	Observational study	RA patients undergoing elective hip/knee arthroplasty	494 arthroplasties	Antirheumatic treatment exposure, including TNF inhibitors	Exposure groups within RA cohort	PJI	E
Chung 2021 [45]	TKA	Retrospective cohort	RA vs. OA patients undergoing TKA	64,341 TKAs: 1126 RA and 63,215 OA	Disease cohort comparison rather than medication-timing study	RA vs. OA	Acute SSI, 90-day and 1-year SSI	S
Cordtz 2018 [46]	THA/TKA	Nationwide cohort study	RA patients undergoing THA/TKA	3913 RA and 120,499 OA	Preoperative treatment exposure, including biologics	Exposure groups within RA cohort	Revision, PJI, mortality	E
Cordtz 2020 [47]	THA/TKA	Register-based cohort study	RA patients undergoing THA/TKA	2899 RA and 112,571 OA	RA arthroplasty cohort; treatment-era complication assessment	RA vs. matched comparators/within-cohort analysis	Medical complications after THA/TKA	S
Di Martino 2023 [48]	THA	Registry-based cohort study	Inflammatory arthritis patients undergoing THA	1464 THAs total: 121 perioperative TNFi, 870 non-bDMARD/tsDMARD inflammatory arthritis, 473 OA controls	Perioperative TNF inhibitor exposure	Non-bDMARD/tsDMARD exposure groups	THA survival, infection, revision	E
Takakubo 2024 [49]	THA/Revision THA	Retrospective cohort	RA patients undergoing THA	204 THAs; revision series also analyzed	Biologic-agent era THA outcomes	RA vs. non-RA THA comparisons/revision analysis	Dislocation, revision, infection	S
Kubota 2014 [50]	Total joint arthroplasty	Retrospective study	RA patients undergoing TJA	567 joints: 267 biologic and 300 non-biologic	Biologic-agent exposure	Biologic vs. non-biologic exposure	Postoperative infection	E
(**B**)								
**Author & Year**	**Surgery Type**	**Study Design**	**Population Studied**	**N (Patients/Procedures)**	**Perioperative Medication Strategy/Exposure**	**Comparator**	**Primary Outcomes**	**Evidence Role**
Barnard 2012 [3]	Elective hand surgery/MCP joint replacement	Observational case series	RA patients undergoing hand surgery	35 hands/140 wounds	Continuation of antirheumatic medications except injectable biologics	None/within-cohort description	Wound healing complications	D
Bibbo 2004 [4]	Elective foot and ankle surgery	Retrospective comparative study	RA patients undergoing foot/ankle surgery	31 patients/141 procedures	TNF-α inhibition therapy	TNF inhibitor exposure vs. no TNF inhibitor exposure	Infectious and healing complications	E
Cathey 2026 [6]	Elective hand surgery	Cohort study	RA patients undergoing elective hand surgery	7929 RA patients	Perioperative biologic DMARD exposure	Biologic-exposed vs. non-exposed groups	Infection and wound complications	E
Klifto 2020 [7]	Elective hand surgery	Retrospective review	RA patients undergoing elective hand surgery	88 cases	Perioperative immunosuppressant medication exposure	Medication-exposed vs. unexposed groups	Overall complications, wound-healing failure, hematoma, SSI	E
Nishida 2026 [16]	Orthopedic surgery	Retrospective case series	RA patients undergoing orthopedic surgery while managed with ozoralizumab	9 patients/12 procedures	Perioperative ozoralizumab discontinuation and restart strategy	None	SSI, DWH, flare	D
Ito 2025 [18]	Orthopedic surgery	Multicenter cohort study	RA patients receiving bDMARDs or tsDMARDs undergoing orthopedic surgery	1953 patients	Perioperative discontinuation of antirheumatic drugs	Continuation vs. discontinuation	SSI, DWH, mortality, flare	D
Grennan 2001 [22]	Elective orthopedic surgery	Randomized controlled study	RA patients undergoing elective orthopedic procedures	388 patients	Methotrexate continuation	Methotrexate withholding/alternative treatment groups	Early postoperative complications, flare	D
den Broeder 2007 [24]	Mixed elective orthopedic surgery	Large retrospective study	RA patients undergoing elective surgery with attention to anti-TNF exposure	768 patients/1219 procedures	Perioperative anti-TNF continuation	Anti-TNF exposure vs. no anti-TNF exposure/within-cohort risk factors	SSI and other postoperative complications	D
Hayata 2011 [26]	Orthopedic surgery	Observational study	RA patients undergoing orthopedic surgery while receiving infliximab	52 procedures	Infliximab exposure and timing to surgery	Within-cohort interval comparisons	Surgical efficacy and complications	D
Kawakami 2010 [27]	Joint surgery	Retrospective comparative study	RA patients treated with TNF blockers undergoing joint surgery	112 patients/128 surgeries	Perioperative interruption of TNF blockers	TNF-blocker group vs. DMARD controls/interruption strata	Postoperative complications, SSI, DVT	D
Momohara 2013 [29]	Orthopedic surgery	Multicenter observational study	RA patients treated with tocilizumab undergoing orthopedic surgery	122 patients/161 cases	Tocilizumab treatment	Internal cohort comparisons	Perioperative clinical features and complications	E
Bekki 2024 [30]	Orthopedic surgery	Retrospective observational study	RA patients treated with JAK inhibitors undergoing orthopedic surgery	62 JAK inhibitor-treated patients + 62 matched bDMARD controls	JAK inhibitor exposure	JAK inhibitor vs. bDMARD-treated patients	Postoperative complications, flare	E
Nishida 2024 [31]	Orthopedic surgery	Retrospective study	RA patients treated with JAK inhibitors undergoing orthopedic surgeries	49 procedures	JAK inhibitor use and interruption interval	Within-cohort interruption strata	Early postoperative complications, flare	D
Kadota 2016 [34]	Orthopedic surgery	Retrospective cohort	RA patients undergoing elective orthopedic surgery	1036 procedures	Multiple perioperative risk factors, including medication exposure	Within-cohort risk-factor comparisons	SSI, DWH	S
Kiso 2025 [35]	Elective orthopedic surgery	Retrospective cohort	RA patients undergoing elective orthopedic surgeries	965 procedures	bDMARD exposure	bDMARD-exposed vs. unexposed/propensity-matched groups	SSI, DWH	E
Kubota 2012 [36]	Elective surgery	Retrospective study	RA patients treated with biologics undergoing elective orthopedic surgery	516 joints/procedures	Biologic therapy exposure	Biologic vs. non-biologic exposure	Perioperative complications, DWH, infection	E
Suzuki 2011 [38]	Orthopedic surgery	Nationwide observational survey	RA patients undergoing orthopedic surgery under biologic or nonbiologic treatment	59,807 procedures overall	Biologic vs. nonbiologic treatment exposure	Biologic vs. nonbiologic groups	Postoperative complications, infection	E
Tada 2016 [39]	Elective orthopedic procedures	Retrospective cohort	RA patients undergoing elective orthopedic procedures	332 procedures	Biologic DMARD exposure	With vs. without biologic exposure	DWH, SSI	E
Yano 2016 [40]	Forefoot surgery	Retrospective cohort	RA patients undergoing forefoot surgery	192 feet	Operative and patient factors, including medication exposure	DWH vs. no DWH/within-cohort analysis	DWH	S
Richter 2018 [42]	Orthopedic surgery utilization in a population-based RA cohort	Population-based cohort study	RA patients followed for orthopedic surgery occurrence	1077 patients	Demographic and disease-related predictors	Within-cohort comparisons	Occurrence and timing of orthopedic surgery; sex differences and temporal trends	S
Yamada 2020 [51]	Mixed orthopedic surgery	Retrospective cohort	RA patients undergoing orthopedic surgery	175 patients	Preoperative biologic/tsDMARD use and surgery type	Within-cohort risk-factor comparisons	Postoperative disease activity, EULAR response	S
Okita 2021 [52]	Orthopedic surgery	Retrospective cohort	RA patients treated with biologics undergoing orthopedic procedures	276 procedures	Biologic therapy, including tocilizumab exposure	Within-cohort risk-factor comparisons	DWH	E
Sakuraba 2022 [53]	Primary cervical spine surgery	Retrospective cohort	RA patients undergoing cervical spine surgery	139 patients	Perioperative risk factors, including glucocorticoid burden	Complication vs. no-complication groups	Perioperative complications, severe events	S

Evidence-role definitions: D, direct perioperative strategy evidence evaluating medication continuation, withholding, interruption duration, restart timing, medication modification, or short-term perioperative administration; E, medication-exposure evidence comparing medication-exposed and unexposed groups, medication classes, or dose strata; S, supportive clinical-context evidence concerning disease status, surgical utilization, patient or procedural risk, operative complexity, or postoperative interpretation. Supportive studies were retained among the 43 original clinical studies but were not used as direct evidence for medication continuation or withholding. Abbreviations: RA, rheumatoid arthritis; THA, total hip arthroplasty; TKA, total knee arthroplasty; TAA, total ankle arthroplasty; TJA, total joint arthroplasty; csDMARD, conventional synthetic disease-modifying antirheumatic drug; bDMARD, biologic disease-modifying antirheumatic drug; tsDMARD, targeted synthetic disease-modifying antirheumatic drug; JAK, Janus kinase; TNF, tumor necrosis factor; SSI, surgical site infection; PJI, periprosthetic joint infection; DWH, delayed wound healing; DVT, deep vein thrombosis; SDAI, simplified disease activity index; EULAR, European Alliance of Associations for Rheumatology.

### 3.3. Quality Assessment

The methodological quality of the included evidence was limited by the predominance of retrospective and non-randomized study designs. Most studies were vulnerable to selection bias, confounding by indication, heterogeneity in perioperative medication-hold intervals, inconsistent definitions of surgical site infection, delayed wound healing and flare, and variable follow-up duration. Large registry and administrative database studies improved power and external validity but often lacked granular information on disease activity, wound status, exact interruption timing, and surgeon-level technical factors. The domain-based quality judgments for the non-randomized evidence base are summarized in Figure 2. The single randomized study was judged to have some concerns overall on RoB 2 assessment, mainly because of limited reporting of the randomization process and potentially selective reporting. For visualization, its overall judgment was harmonized with the categories used for the non-randomized studies in Figure 2.

### 3.4. Primary Outcome Synthesis

Within each primary outcome domain, evidence is presented according to its directness for the medication-management question. Direct perioperative strategy evidence is discussed first, followed by medication-exposure evidence and then supportive risk-modifier evidence. Medication recommendations are based only on direct strategy evidence and cautious interpretation of exposure studies. Supportive risk factors are presented to explain confounding and clinical heterogeneity and are not interpreted as evidence for continuation or withholding.

#### 3.4.1. Surgical Site Infection and Periprosthetic Joint Infection

Direct perioperative strategy evidence. Studies directly evaluating continuation, discontinuation, or interruption timing did not demonstrate a consistent infection-related benefit from longer medication withholding. Den Broeder et al. reported that perioperative anti-tumor necrosis factor (TNF) continuation was not significantly associated with surgical site infection (SSI; adjusted OR 1.50), whereas previous skin or wound infection, elbow surgery, and foot and ankle surgery were more strongly associated with infection risk [24]. Similarly, among patients receiving infliximab, an interval of less than 4 weeks between the last infusion and hip or knee arthroplasty was not associated with a greater risk of 30-day serious infection or 1-year periprosthetic joint infection (PJI) compared with an interval of 8–12 weeks [25]. In the multicenter cohort of 1953 patients receiving biologic disease-modifying antirheumatic drugs (bDMARDs) or targeted synthetic disease-modifying antirheumatic drugs (tsDMARDs), perioperative treatment discontinuation did not significantly reduce SSI or mortality [18].

Direct evidence concerning newer targeted therapies was limited by small sample sizes. Nishida et al. observed no SSI among 49 orthopedic procedures in patients receiving Janus kinase (JAK) inhibitors, although interruption intervals varied within the cohort [31]. Similarly, the ozoralizumab case series reported no SSI among 12 procedures performed using a defined discontinuation and postoperative restart strategy; however, the absence of a comparator group and the small sample precluded conclusions regarding the superiority of this approach [16]. Overall, the direct strategy evidence did not demonstrate that progressively longer interruption consistently reduced postoperative infection.

Medication-exposure evidence. Findings from medication-exposed versus unexposed comparisons were heterogeneous. In the Japanese Orthopaedic Association survey, infection occurred in 46 of 3468 procedures performed under biologic treatment (1.3%) and in 567 of 56,339 procedures performed under nonbiologic treatment (1.0%). In the joint arthroplasty subgroup, infection rates were 2.1% and 1.0%, respectively, corresponding to an OR of 2.12 (95% CI 1.48–3.03) for biologic-treated procedures [38]. Momohara et al. similarly reported acute SSI after total hip or knee arthroplasty in 24 of 420 procedures (5.7%) and organ/space SSI in 3 procedures (0.7%); biologic DMARD exposure was associated with higher acute SSI risk (OR 5.69, 95% CI 2.07–15.61), particularly with infliximab and etanercept [37].

Other exposure-based studies did not identify a comparable infection signal. Borgas et al. reported an overall PJI rate of 1.4% among 494 elective hip and knee arthroplasties, including 2.8% after total knee arthroplasty and 0% after total hip arthroplasty. Only 1 of 157 patients receiving a TNF inhibitor developed PJI, and no clear association between TNF inhibitor exposure and infection was identified [44]. In the Danish nationwide cohort, RA was associated with greater PJI and mortality risk, but preoperative bDMARD exposure within 90 days was not significantly associated with revision, PJI, or death [46]. Kiso et al. identified SSI in 12 of 965 procedures, including 3 infections among 414 bDMARD-exposed procedures, with no significant exposure-related difference after propensity-score matching [35]. Bekki et al. also found no significant difference in postoperative complications other than flare between patients treated with JAK inhibitors and those treated with bDMARDs [30].

In the large United States arthroplasty cohort of 10,923 procedures, postoperative hospitalized infection and 1-year PJI were broadly similar across the biologic agents evaluated [20]. In contrast, chronic baseline glucocorticoid exposure showed a dose-related association with adverse outcomes. A daily dose greater than 10 mg was associated with a predicted hospitalized infection risk of 13.25%, compared with 6.78% among patients not receiving glucocorticoids, and a predicted 1-year PJI incidence of 3.83%, compared with 2.09% without glucocorticoids [20]. These findings concern chronic baseline exposure and should not be extrapolated to short-term perioperative glucocorticoid administration.

Supportive clinical-context and risk-modifier evidence. Several studies indicated that infection risk was influenced by disease-, patient-, and procedure-related factors in addition to medication exposure. Bongartz et al. observed PJI in 23 of 657 arthroplasties (3.7%), with revision arthroplasty and previous PJI identified as major predictors [43]. Chung et al. reported acute SSI rates of 2.58% among patients with RA and 2.66% among patients with osteoarthritis undergoing total knee arthroplasty, without a significant difference in 90-day or 1-year infection risk [45]. A related Danish analysis found that RA was associated with a higher risk of nonsurgical infection after total hip or knee arthroplasty, while biologic-treated patients with RA did not demonstrate a clear excess infection risk despite higher estimates for some secondary outcomes [47].

Tada et al. found no independent medication-related increase in SSI among 332 elective orthopedic procedures. Instead, older age (OR 1.11), longer operative duration (OR 1.02), and a preoperative white blood cell count greater than 10,000/μL (OR 3.66) were associated with SSI [39]. The infliximab-timing study also identified chronic baseline glucocorticoid doses greater than 10 mg/day as being associated with increased 30-day infection and 1-year PJI risk, despite finding no significant association with shorter infliximab interruption [25]. These supportive findings demonstrate important sources of confounding and postoperative risk but do not directly establish whether a particular antirheumatic medication should be continued or withheld.

Taken together, the direct perioperative strategy and medication-exposure evidence did not demonstrate a consistent reduction in SSI or PJI with longer interruption of biologic or targeted synthetic therapy. Chronic baseline glucocorticoid exposure, previous infection, revision surgery, operative duration, anatomical site, and other patient or procedure-related factors frequently influenced infection risk. However, these risk-modifier associations should not be interpreted as direct evidence for a specific medication continuation, withholding, or restart strategy.

#### 3.4.2. Delayed Wound Healing

Delayed wound healing emerged as a major outcome in non-arthroplasty and foot/ankle literature. In the largest forefoot-specific cohort, Yano et al. reported delayed wound healing in 40 of 192 feet (20.8%), and multivariable analysis identified operative time as the sole independent predictor, with an OR of 1.19 per 10-min increase [40]. A similar theme appeared in the TAA comparative study by Yi et al., in which delayed wound healing >4 weeks occurred in 0 of 18 patients managed with perioperative medication modification versus 4 of 16 (25%) patients without modification (*p* = 0.039). Weekly wound assessment also favored the medication-modified group, with ASEPSIS scores of 13.8 vs. 17.1 at 2 weeks, 9.2 vs. 14.7 at 3 weeks, and 3.1 vs. 6.8 at 4 weeks [33]. These findings indicate that wound outcomes may be especially procedure sensitive and are not adequately represented by arthroplasty-only guidance.

Across mixed orthopedic cohorts, delayed wound healing was not consistently linked to biologic exposure. Tada et al. found that no DMARD class independently predicted delayed wound healing, whereas advanced age (OR 1.16), prolonged surgery (OR 1.02), preoperative WBC > 10,000/μL (OR 4.56), and foot surgery (OR 6.60) were significant risk factors [39]. Kiso et al. likewise found no association between bDMARD use and delayed wound healing in 965 elective procedures, although age, diabetes mellitus, foot and ankle surgery, and prior musculoskeletal infection remained important predictors [35]. In earlier biologic-era cohorts, Kubota et al. reported delayed wound healing in 15 of 235 biologic-exposed joints (6.4%) versus 12 of 281 non-biologic joints (4.3%), without a statistically significant difference; even stratification by etanercept dose or shorter versus longer waiting interval before surgery did not materially change this pattern [36].

More specialized cohorts suggested that drug class might still interact with local wound biology in selected settings. In 276 orthopedic procedures performed in RA patients already treated with biologics, Okita et al. specifically examined delayed wound healing and concluded that foot and ankle surgery and tocilizumab exposure warranted particular caution [52]. In JAK inhibitor studies, delayed wound healing was rare: Nishida et al. documented 1 case of delayed wound healing in 49 procedures [31], while Bekki et al. found no association between longer withholding duration and delayed wound healing [30]. The 2025 multicenter discontinuation analysis reinforced the same pattern, reporting no significant reduction in delayed wound healing with perioperative discontinuation of bDMARDs or tsDMARDs [18]. Taken together, direct evidence concerning the effect of perioperative medication interruption on delayed wound healing remained limited and inconsistent. Medication-exposure studies did not demonstrate a uniform increase in delayed wound healing associated with biologic or targeted synthetic therapy across orthopedic settings, and longer treatment interruption did not consistently reduce this outcome [18,30,31,35,36]. Supportive studies more consistently identified anatomical site, particularly foot and ankle surgery, operative duration, age, diabetes mellitus, previous musculoskeletal infection, and other procedure-related factors as relevant risk modifiers [35,39,40,52]. These findings indicate that wound-healing outcomes are strongly influenced by patient- and procedure-specific factors; however, they do not establish that continuation or withholding of a specific antirheumatic medication directly causes or prevents delayed wound healing.

#### 3.4.3. Postoperative Rheumatoid Arthritis Flare

Compared with infection and wound complications, flare was less consistently reported in older studies but emerged as a major endpoint in contemporary cohorts. The strongest modern signal came from the multicenter orthopedic study by Ito et al., in which perioperative discontinuation of biologic or targeted synthetic therapy did not reduce SSI, delayed wound healing, or mortality, but was associated with a substantially higher incidence of postoperative flare, particularly in patients receiving interleukin-6 inhibitors [18]. Bekki et al. similarly found that JAK inhibitor-treated patients had a higher rate of postoperative flare than bDMARD-treated patients (29.0% vs. 12.1%, *p* = 0.01), and that a total drug-withholding period of ≥11 days was associated with more flares [30]. Nishida et al. observed flare in 2 patients after 3 and 9 days of JAK inhibitor discontinuation [31]. These studies collectively argue that the cost of prolonged preoperative interruption may be most visible in postoperative disease control rather than in infection prevention.

Flare remained uncommon but clearly possible in smaller contemporary series. In the ozoralizumab case series, 1 of 9 patients experienced flare before the drug was restarted despite the absence of SSI or delayed wound healing [16]. By contrast, no flare occurred during steroid adjustment in the TAA medication-modification study [33], although this likely reflects both small sample size and structured perioperative coordination. In older wound-focused cohorts, flare was often not systematically captured, limiting comparison with the newer literature. Taken together, postoperative flare was most clearly evaluated in recent observational studies comparing continuation with discontinuation or examining different interruption durations. These studies suggest that longer withholding of biologic or targeted synthetic therapy may be associated with an increased risk of postoperative flare [18,30,31]. However, the available evidence does not establish causality or identify a single optimal interruption or restart interval because flare definitions, medication classes, surgical procedures, and withholding durations were heterogeneous. Postoperative flare should therefore be considered alongside infection and wound-healing outcomes when applying individualized, guideline-informed perioperative medication strategies.

### 3.5. Secondary Outcome Synthesis

#### 3.5.1. Reoperation, Revision, and Readmission

Secondary outcome data were more common in arthroplasty cohorts than in mixed orthopedic studies. In the nationwide Japanese THA study, RA was independently associated with reoperation during hospitalization, with an OR of 2.254 (95% CI 1.687–3.013), even though the same analysis did not show a significant excess of postoperative infection or periprosthetic fracture [21]. Takakubo et al. similarly emphasized that, despite improved medical therapy in the biologic and JAK inhibitor era, revision THA in RA continued to occur for aseptic loosening, infection, and frequent dislocation; among 561 revision THAs, 16 (2.9%) occurred in RA patients, with 81.3% attributed to aseptic loosening and 12.5% to infection [49]. In contrast, the TAA comparative study found no significant difference in reoperation between patients managed with and without perioperative medication modification, with rates of 11.1% versus 6.3% at 2 years [33]. These findings suggest that the effect of perioperative medication strategy on revision-related endpoints is less consistent than its effect on early wound outcomes and may be strongly influenced by implant mechanics, bone quality, and baseline joint destruction. Longer-term THA data were also reassuring for perioperative TNF inhibitor exposure: in a registry-based inflammatory arthritis cohort, revision occurred in 2.5% of perioperative TNFi users versus 3.0% of non-bDMARD/tsDMARD patients, with no significant difference in implant survival or postoperative infection risk [48]. In total ankle arthroplasty, 48 of 50 ankles avoided revision, although talar component subsidence remained an important mechanical issue, particularly in biologic-treated cases [5].

Readmission outcomes were reported in only a subset of medication-focused studies. In the large U.S. biologic/glucocorticoid arthroplasty cohort, risks of hospitalized infection, prosthetic joint infection, and 30-day readmission were broadly similar across biologic agents, whereas glucocorticoid exposure showed a dose-dependent association with worse postoperative outcomes [20]. By contrast, in the 2024 glucocorticoid arthroplasty cohort of 849 RA patients, perioperative short-term glucocorticoid administration was not associated with higher 90-day readmission and was accompanied by lower rescue opioid use (17.9% vs. 44.6%, *p* < 0.001) and lower opioid consumption (4.7 ± 2.1 mg vs. 8.9 ± 4.6 mg, *p* < 0.001) [32]. These data suggest that perioperative glucocorticoid exposure needs to be distinguished carefully by context: chronic baseline glucocorticoid burden appeared harmful, whereas short perioperative glucocorticoid use in selected non-chronic users did not worsen short-term readmission metrics [20,32].

#### 3.5.2. Dislocation and Mechanical Complications

Mechanical complications were particularly prominent in hip arthroplasty studies. In the THA disease-activity cohort, dislocation occurred in 15 of 337 RA patients (4.5%), and rates increased significantly across disease-activity strata, with higher mean SDAI associated with greater overall complication risk [19]. In the nationwide Japanese analysis, RA independently increased dislocation risk with an OR of 2.783 (95% CI 1.641–4.720) [21]. Takakubo et al. likewise reported a dislocation rate of 2.9% (6/204) in RA-THA compared with 1.0% in non-RA THA, confirming that instability remained a distinct unmet need in contemporary RA hip arthroplasty [49]. Together, these findings support the view that mechanical outcomes in RA are not captured adequately by infection-centered perioperative frameworks alone.

Outside THA, mechanical and local surgical complications were highly procedure specific. In the TAA study, postoperative events included marginal skin necrosis, malleolar fracture, tibial component malalignment, talar subsidence, heterotopic ossification, soft-tissue impingement, and progression of adjacent-joint arthritis, but these did not differ significantly between medication-modified and unmodified groups apart from delayed wound healing [33]. In the elective hand-surgery cohort, overall complications occurred in 8 of 88 cases (9%) and included wound-healing failure, tendon rupture, hematoma, and surgical-site infection, with no statistically significant difference between medication-exposed and unexposed groups [7]. In the older infliximab orthopedic series, only 2 of 52 procedures (3.8%) were complicated by signs of infection or surgical events requiring attention, and no relationship was found between complication occurrence and timing from infliximab infusion to surgery [26]. These observations indicate that once the analysis moves beyond large-joint arthroplasty, complication phenotypes become more locally anatomical and less easily reduced to a single composite endpoint.

#### 3.5.3. Mortality, Venous Thromboembolism, and Other Outcomes

Mortality was infrequently reported and rarely event-rich enough for strong inference. In the large glucocorticoid arthroplasty cohort, 30-day mortality did not differ between patients who did and did not receive perioperative glucocorticoids [32]. Similarly, the nationwide THA study found no significant increase in in-hospital mortality attributable to RA despite an odds ratio of 2.254 that did not meet the study’s significance threshold, and RA was not a significant predictor of pneumonia, pulmonary embolism, or sepsis in multivariable analyses [21]. These findings are broadly consistent with the view that short-term mortality after modern arthroplasty in RA is uncommon and may be less sensitive to perioperative medication strategy than infection, wound, or instability outcomes.

Venous thromboembolism outcomes were inconsistently reported and difficult to compare across studies. In the nationwide Japanese THA cohort, deep vein thrombosis was paradoxically lower in RA than OA after matching, with an OR of 0.524 (95% CI 0.436–0.630), while pulmonary embolism showed no significant difference [22]. The 2017 ACR/AAHKS guideline explicitly noted that it did not address perioperative venous thromboembolism prophylaxis and cautioned against extrapolating THA/TKA-focused medication guidance to other orthopedic procedures [11]. Accordingly, the current evidence set provides limited support for any medication-specific conclusion regarding venous thromboembolism in RA orthopedic surgery.

### 3.6. Factors Modifying Postoperative Risk

#### 3.6.1. Medication Class

The most consistent medication-class signal concerned the relative safety of continuing conventional synthetic DMARDs, especially methotrexate. In the randomized methotrexate study by Grennan et al., 388 RA patients undergoing elective orthopedic procedures showed no increase in postoperative complications with perioperative methotrexate continuation [22]. Barnard et al. also reported favorable wound outcomes in 35 hands (140 wounds) undergoing metacarpophalangeal joint replacement when antirheumatic medications were continued perioperatively except for injectable biologics, with no wound dehiscence or deep infection and only one minor superficial infection [3]. These findings align with guideline recommendations supporting csDMARD continuation rather than routine cessation [11,12].

Biologic therapy showed a more heterogeneous pattern. Older anti-TNF observational studies ranged from apparently safe continuation in elective foot and ankle surgery [4] to higher arthroplasty infection odds in some biologic-era cohorts [37,38]. In the Japanese Orthopaedic Association survey, SSI occurred in 1.3% of biologic-treated procedures overall and 2.1% of biologic-treated joint arthroplasties, compared with 1.0% under nonbiologic treatment in the arthroplasty subgroup [38]. However, more recent timing studies summarized in the 2022 ACR/AAHKS guideline found no difference in postoperative outcomes when infliximab or abatacept were interrupted for approximately one dosing interval versus longer holds [12]. Hand-surgery data also did not show an excess of overall complications in patients exposed to perioperative immunosuppressive therapy [7]. Thus, the modern evidence does not support a uniform assumption that longer biologic withdrawal meaningfully improves postoperative outcomes across orthopedic procedures.

Targeted synthetic therapies remain supported by smaller but increasingly relevant datasets. The orthopedic JAK inhibitor studies showed low SSI and delayed wound-healing rates [30,31], while the ozoralizumab case series reported no SSI or delayed wound healing across 12 procedures but did document one flare before treatment restart [16]. These emerging data favor individualized interruption strategies rather than prolonged empiric cessation, although evidence remains too sparse to support definitive drug-specific conclusions.

#### 3.6.2. Surgery Type

Procedure type consistently modified risk. Arthroplasty cohorts were dominated by infection, dislocation, and revision-related outcomes, whereas forefoot and ankle studies emphasized delayed wound healing and local soft-tissue complications [4,5,33,40]. Hand-surgery studies highlighted lower absolute infection rates but remained concerned about wound breakdown and tendon-related failure [3,7]. Spine surgery introduced a different complication profile, with severe events linked to fusion extent and systemic frailty rather than any single drug class [53]. This heterogeneity supports the caution expressed in the 2017 ACR/AAHKS guideline that THA/TKA-based perioperative recommendations should not be freely extrapolated to other orthopedic procedures [11].

#### 3.6.3. Disease Activity, Steroid Burden, Recent Arthroplasty or Revision, and Operative Complexity

Non-medication factors were among the strongest and most consistent determinants of postoperative outcome. Kadota et al. identified SSI in 19 and DWH in 15 of 1036 elective procedures; foot and ankle surgery increased SSI risk, while TKA and longer disease duration increased delayed wound-healing risk [34]. In contrast, Kawakami et al. reported a stronger anti-TNF-related signal in major orthopedic surgery, with SSI in 12.5% of TNF-blocker procedures versus 2% in DMARD controls and postoperative DVT in 51% versus 26%, respectively [27]. Kubota et al. later found no significant increase in postoperative infection after total joint arthroplasty with biologic use and identified foot/ankle surgery, rather than biologic exposure itself, as the dominant infection predictor [50]. In THA, each 1-unit increase in mean SDAI was associated with higher complication risk (OR 1.015) [19]. In TNF-blocker-exposed arthroplasty patients, Gilson et al. found two major infection predictors: primary TJA or revision of the subsequently infected joint within the previous year (OR 88.3, 95% CI 1.1–7071.0) and daily steroid intake (OR 5.0 per 5 mg/day increase, 95% CI 1.1–21.6) [17]. In the large U.S. arthroplasty cohort, chronic glucocorticoid exposure above 10 mg/day was associated with markedly higher predicted risks of hospitalized infection and prosthetic joint infection [20]. In forefoot surgery, operative time was independently associated with delayed wound healing (OR 1.19 per 10 min) [40]. These studies collectively suggest that postoperative risk in RA is frequently driven by disease activity, steroid burden, recency and complexity of prior surgery, and operative intensity at least as much as by perioperative drug-hold strategy itself.

#### 3.6.4. Distinction Between Chronic Baseline, Short-Term Perioperative, and Stress-Dose Glucocorticoid Exposure

Glucocorticoid exposure was divided into three clinically distinct categories: chronic baseline therapy, short-term perioperative administration, and perioperative stress-dose supplementation.

Chronic baseline exposure: Several observational studies evaluated the patient’s established daily glucocorticoid exposure before surgery. In TNF-blocker-exposed arthroplasty cohorts, higher baseline doses were associated with greater prosthetic infection risk [17]. In the large U.S. arthroplasty cohort, chronic glucocorticoid exposure showed a dose-related association with hospitalized infection, periprosthetic joint infection, and readmission, with the greatest risks among patients receiving more than 10 mg/day [20]. Other risk-factor studies also identified glucocorticoid burden as a relevant marker of postoperative risk [25,41,53]. Because these findings were observational, residual confounding by disease severity and comorbidity remained possible.

Short-term perioperative administration: Lai et al. evaluated a brief perioperative glucocorticoid regimen in patients undergoing total joint arthroplasty [32]. Short-term administration was not associated with increased early complications or 90-day readmission and was associated with reduced postoperative opioid requirements. This exposure was clinically different from chronic baseline therapy and should not be interpreted using the same risk estimates.

Stress-dose supplementation: No included study directly compared perioperative stress-dose supplementation with continuation of the usual baseline dose or with no supplementation. Therefore, the present review cannot determine the clinical benefits or risks of stress-dose supplementation. Recommendations concerning adrenal supplementation must be attributed to separate endocrine, anesthetic, or perioperative guidelines rather than to the evidence included in this review.

### 3.7. Overall Certainty and Consistency of the Evidence

Overall certainty of the evidence was limited. Most included studies were retrospective, nonrandomized, and vulnerable to confounding by indication, especially because patients with higher disease activity, greater glucocorticoid exposure, prior infection, or more complex surgery were often also those most likely to receive biologics or undergo perioperative treatment interruption [17,18,19,20]. Outcome definitions were heterogeneous, particularly for delayed wound healing and flare, and procedure types ranged from THA/TKA to foot, ankle, hand, and spine surgery, reducing the appropriateness of broad pooled inference [7,33,40].

Despite these limitations, several directional findings were reasonably consistent across the literature. The available randomized evidence indicated that methotrexate continuation was not associated with an evident increase in early postoperative complications [22], whereas direct comparative evidence for other csDMARDs remained limited [23]. Observational strategy and medication-exposure studies did not consistently demonstrate that longer interruption of biologic or targeted synthetic therapy reduced infection or delayed wound-healing risk, while postoperative flare emerged as an important competing consequence of treatment interruption [16,18,25,28,30,31]. Supportive clinical-context studies identified chronic baseline glucocorticoid burden, disease activity, previous infection or revision, anatomical site, and operative complexity as relevant risk modifiers [17,19,20,34,40,43,53]. These supportive associations were not interpreted as direct evidence for a medication continuation or withholding strategy. Using the GRADE approach, the certainty of evidence for all three primary outcomes was judged to be very low. Certainty for the key secondary outcome domains was also judged to be very low because the evidence base was predominantly observational, clinically heterogeneous, and variably reported across procedure types, medication classes, and outcome definitions. A summary of certainty judgments for primary and secondary outcomes is presented in Table 2.

### 3.8. Reporting Bias Assessment

Formal outcome-level assessment of reporting bias was limited because meta-analysis was not performed and the evidence base was dominated by retrospective observational studies with heterogeneous outcome definitions. Nevertheless, incomplete outcome reporting and selective emphasis on infection endpoints over wound-healing and flare outcomes were recurrent concerns across the literature. These considerations contributed to downgrading certainty in the GRADE assessment, although publication bias could not be confirmed formally.

## 4. Discussion

### 4.1. Principal Findings

This systematic review retained 43 original clinical studies concerning perioperative antirheumatic medication management and its broader clinical context in adults with RA undergoing orthopedic surgery. Six non-original sources were used only to frame guideline recommendations, previous syntheses, and research gaps. To address variation in evidence directness, the original studies were classified as direct perioperative strategy evidence, medication-exposure evidence, or supportive clinical-context evidence. Supportive studies, including evidence concerning surgical utilization and postoperative inflammatory-marker interpretation, were not used to formulate causal medication recommendations.

Three medication-related findings emerged. First, methotrexate continuation was not associated with an evident increase in early postoperative complications in the available randomized evidence, although direct evidence for other csDMARDs was limited [22,23]. Second, observational studies of biologic and targeted synthetic therapies did not demonstrate a consistent reduction in infection or delayed wound healing with longer interruption, while several contemporary studies associated treatment interruption with postoperative flare [16,18,25,28,30,31]. Third, chronic baseline glucocorticoid exposure showed dose-related associations with adverse postoperative outcomes, but it represented a different exposure from short-term perioperative glucocorticoid administration [17,20,32]. No included study directly evaluated stress-dose supplementation.

Supportive studies separately demonstrated that disease activity, previous infection or revision, anatomical site, operative duration, procedure type, and surgical complexity may influence postoperative outcomes [19,21,34,40,43,53]. These findings explain important sources of confounding and heterogeneity but do not directly establish the optimal timing of antirheumatic therapy.

### 4.2. Interpretation of the Infection–Wound Healing–Flare Tradeoff

The dominant clinical question in perioperative RA care is not simply whether immunosuppression increases complications, but whether stopping therapy reduces those complications enough to justify the risk of flare. The present review indicates that this tradeoff is more nuanced than older infection-centered frameworks imply. Several studies identified postoperative infection signals among biologic-treated patients, particularly in arthroplasty cohorts [17,37,38]. However, these findings were not uniform, and more recent studies suggest that the incremental effect of perioperative drug interruption may be smaller than previously assumed, especially when compared with the influence of host and surgical factors [18,20,35,44]. In the largest recent multicenter study, discontinuation of biologic or targeted synthetic therapy did not reduce surgical site infection, delay wound healing, nor mortality, yet flare was substantially more frequent after interruption [18]. JAK inhibitor studies reported low absolute infection and wound-healing event rates, but flares became more common as withholding periods lengthened [30,31]. These findings question whether progressively longer withholding consistently improves postoperative outcomes. However, because most comparisons were observational and heterogeneous, they do not establish an optimal interruption interval. The evidence instead indicates that infection, wound healing, flare, and rehabilitation consequences should all be considered when applying guideline-based timing recommendations to an individual patient.

### 4.3. Medication Effects Should Be Interpreted in Context, Not in Isolation

The most direct medication evidence in this review concerned methotrexate continuation. In the randomized study by Grennan et al., perioperative continuation of methotrexate was not associated with an increase in early postoperative complications among patients with RA undergoing elective orthopedic surgery [22]. However, direct comparative evidence for other conventional synthetic disease-modifying antirheumatic drugs was substantially more limited. Current recommendations supporting continuation of selected conventional synthetic agents are also reflected in the ACR/AAHKS guidelines [11,12], but these guideline recommendations should not be interpreted as equivalent to direct comparative evidence for every conventional synthetic agent or every type of orthopedic procedure.

Evidence concerning biologic therapy was more heterogeneous. Older observational studies of tumor necrosis factor inhibitors reported conflicting findings, ranging from no evident increase in complications with continued exposure to higher postoperative infection rates among biologic-treated patients [4,17,27,37,38]. These differences may reflect variations in study design, patient selection, disease severity, glucocorticoid exposure, surgical setting, and definitions of postoperative infection.

Later timing-based studies provided more direct evidence concerning perioperative interruption. Shorter preoperative infliximab interruption was not associated with a higher risk of serious infection or periprosthetic joint infection compared with longer interruption, and a similar study of abatacept did not demonstrate a clear postoperative benefit from withholding the drug for a longer period [25,28]. These findings do not establish that biologic therapy should always be continued, but they question whether progressively longer interruption consistently improves postoperative safety. Specific withholding and restart intervals remain informed substantially by clinical guidelines and expert interpretation rather than by high-certainty comparative evidence [11,12].

Evidence concerning targeted synthetic therapies and newer biologic agents remained limited. Small observational studies of Janus kinase inhibitors reported low absolute rates of surgical site infection and delayed wound healing, while longer treatment interruption was associated with more postoperative flare in some analyses [30,31]. The small ozoralizumab case series similarly reported no surgical site infection or delayed wound healing, although its limited sample size and absence of a comparator group prevent firm conclusions regarding safety or optimal interruption timing [16]. Therefore, these studies do not currently demonstrate a strong or consistent increase in early infectious or wound-healing complications, but they are insufficient to establish a definitive perioperative strategy.

Two non-original contextual evidence syntheses further illustrate the uncertainty of the field. The evidence review supporting the Japan College of Rheumatology recommendations reported an overall association between biologic exposure and surgical site infection but not delayed wound healing, while also identifying age, glucocorticoid exposure, and foot, ankle, or spine surgery as relevant risk factors [13]. Mabille et al. reported an association between perioperative anti-TNF exposure and postoperative infection, but preventive treatment discontinuation did not significantly reduce that risk [14]. These sources were used to place the original clinical studies in context and were not treated as direct patient-level evidence in the present review.

Taken together, the included studies do not justify a universal prolonged-withholding strategy across all biologic and targeted synthetic therapies. Medication decisions should instead be interpreted according to the specific agent, the directness and certainty of the available evidence, baseline disease control, chronic glucocorticoid exposure, previous infection, and the surgical procedure. Detailed interruption and restart schedules should be identified explicitly as guideline-derived recommendations when they are not directly established by the included comparative studies.

### 4.4. Host and Surgical Factors May Matter More than the Hold Interval

Supportive observational studies repeatedly identified disease-, patient-, and surgical-level factors associated with postoperative risk. In arthroplasty, higher disease activity was associated with more complications after THA, with complication rates increasing from remission to high disease activity and mean SDAI independently associated with postoperative risk [19]. In TNF-blocker-exposed arthroplasty patients, recent primary or revision arthroplasty of the subsequently infected joint and higher daily steroid exposure were the main predictors of prosthetic infection [17]. In forefoot surgery, delayed wound healing was driven primarily by operative time rather than by perioperative medication use [40]. In total ankle arthroplasty, structured medication modification appeared beneficial for wound healing, but that benefit occurred in a context where local soft-tissue risk is inherently greater than in large-joint arthroplasty [33]. These findings collectively suggest that the apparent “effect” of medication strategy in many observational studies may partly reflect confounding by disease severity, steroid burden, anatomical site, and surgical complexity. These risk-factor associations are clinically important for adjustment, stratification, and interpretation, but they should not be considered direct evidence concerning medication continuation or withholding. In particular, an observed association between disease activity, surgical complexity, or anatomical site and postoperative complications does not establish that modifying medication timing will reduce that risk.

### 4.5. Generalizability Across Orthopedic Procedures

A major contribution of this review is showing why THA/TKA-derived guidance cannot be assumed to apply uniformly across the full spectrum of orthopedic surgery. The ACR/AAHKS perioperative recommendations were intentionally developed for elective THA/TKA and are conditional because direct high-quality data remain limited [11,12]. However, the orthopedic RA literature extends beyond arthroplasty into foot and ankle surgery, hand surgery, spine surgery, and mixed reconstructive cohorts. These procedures differ substantially in soft-tissue envelope, contamination risk, wound tension, implant dependence, and flare consequences [7,8,33,40,53]. For example, forefoot and ankle studies were dominated by delayed wound healing and local wound-care issues [33,40], whereas THA studies highlighted dislocation, reoperation, and revision [19,21,49], and hand surgery showed a different complication pattern with low infection burden but meaningful wound and tendon-related events [7]. These differences likely explain why the same medication class may appear “safe” in one orthopedic setting and more concerning in another without the findings being truly contradictory. The extension of THA/TKA recommendations to hand, foot, ankle, spine, and other orthopedic procedures therefore represents cautious clinical extrapolation rather than a directly validated medication-management strategy. Procedure-specific evidence should be identified explicitly whenever available, and guideline recommendations developed for elective THA/TKA should not be presented as though they were tested across all orthopedic procedures.

### 4.6. Evidence-Supported Findings and Guideline-Derived Clinical Implications

#### 4.6.1. Findings Supported Directly by the Included Studies

The direct evidence supports a limited number of medication-related conclusions. Methotrexate continuation was not associated with an evident increase in early postoperative complications in the available randomized study, while direct evidence for other csDMARDs was sparse [22,23]. For biologic and targeted synthetic therapies, observational continuation, exposure, and timing studies did not consistently demonstrate that longer interruption reduced SSI, PJI, or delayed wound healing [18,24,25,28,30,31]. Several recent studies associated longer treatment interruption with a greater risk of postoperative flare, although optimal drug-specific interruption intervals could not be established [18,30,31].

Chronic baseline glucocorticoid exposure showed dose-related associations with infection and other postoperative complications [17,20,25]. By contrast, one observational arthroplasty study found no increase in early complications with short-term perioperative glucocorticoid administration [33]. These exposures should not be combined. Stress-dose supplementation was not directly evaluated.

Beyond these early postoperative outcomes, chronic glucocorticoid therapy is also associated with cumulative late skeletal complications, particularly osteoporosis and bone fragility [9]. These long-term effects are clinically relevant because they may necessitate gentle intraoperative positioning and careful postoperative mobilization. They should be considered separately from the effects of brief perioperative glucocorticoid administration.

#### 4.6.2. Recommendations Derived from Guidelines and Clinical Interpretation

Detailed recommendations concerning when to withhold and restart biologic or targeted synthetic therapies derive substantially from current clinical guidelines and expert interpretation rather than from high-certainty comparative evidence in the included studies [11,12]. The ACR/AAHKS recommendations were developed specifically for elective THA and TKA, and their application to hand, foot, ankle, spine, or other procedures should be described as extrapolation.

**Figure 3:**
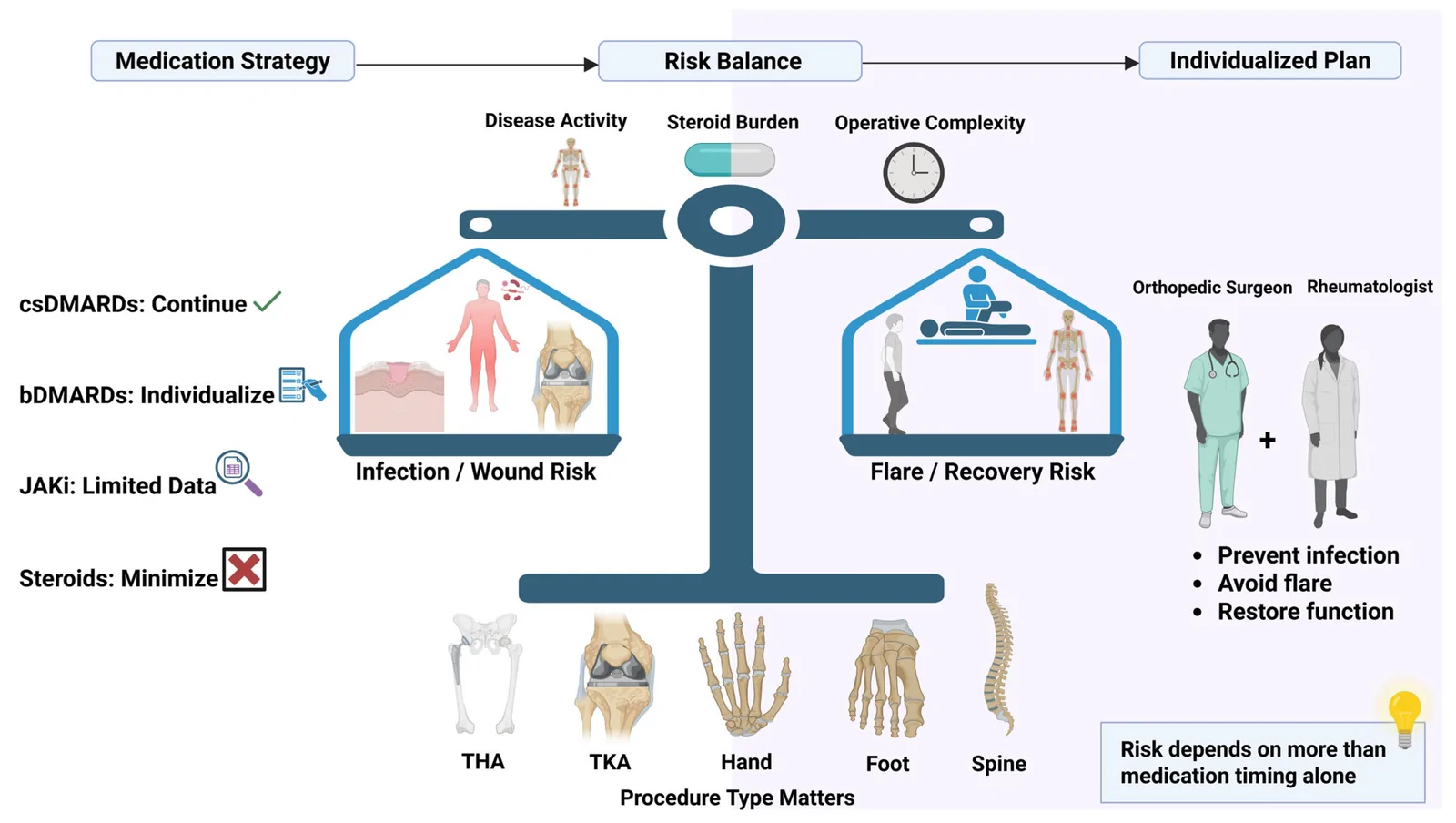
Conceptual framework for perioperative antirheumatic medication decision making in rheumatoid arthritis (RA) patients undergoing orthopedic surgery. Medication strategy should be balanced against infection/wound-healing risk and flare/rehabilitation risk, while accounting for disease activity, chronic glucocorticoid burden, operative complexity, and procedure type. The framework emphasizes individualized orthopedic–rheumatology planning rather than relying on medication timing alone. This figure is an interpretive framework integrating direct medication evidence, supportive clinical-context evidence, and current guideline context; it is not a validated clinical decision rule and does not specify evidence-proven medication interruption or restart intervals. **Abbreviations:** RA, rheumatoid arthritis; csDMARDs, conventional synthetic disease-modifying antirheumatic drugs; bDMARDs, biologic disease-modifying antirheumatic drugs; JAKi, Janus kinase inhibitors; THA, total hip arthroplasty; TKA, total knee arthroplasty. Created in BioRender. alghoul, W. (2026) https://BioRender.com/lpihafo.

Clinical planning may reasonably consider disease control, baseline glucocorticoid exposure, previous infection, revision status, procedure-specific wound risk, and the functional consequences of flare. These considerations support multidisciplinary planning among rheumatology, orthopedic surgery, anesthesia, and the patient. However, the present evidence does not validate a universal medication-timing algorithm or prove that changing any single risk factor will prevent postoperative complications. Table 3 summarizes the evidence by medication class and distinguishes study-supported findings from guideline-derived interpretation. The interactions between medication-related evidence, patient- and procedure-specific risk modifiers, and the competing risks of infection or wound complications and postoperative flare are summarized in Figure 3.

### 4.7. Guideline Scope and Procedure-Specific Interpretation

The ACR/AAHKS guidelines remain the most influential framework for perioperative antirheumatic medication management, but their direct scope is elective THA and TKA in patients with inflammatory arthritis or SLE [11,12]. Therefore, extrapolation to hand, foot, ankle, and spine procedures should be cautious because procedure-specific wound burden, implant dependence, soft-tissue conditions, and rehabilitation priorities differ across orthopedic subspecialties [8,11,12].

### 4.8. Strengths and Limitations

A principal strength of this review is its RA-specific orthopedic focus across arthroplasty and non-arthroplasty procedures, including contemporary evidence concerning JAK inhibitors and newer biologic agents. The review also considered infection, delayed wound healing, postoperative flare, and procedure-specific complications rather than restricting the synthesis to infection alone.

Several limitations should be considered. The evidence was dominated by retrospective observational studies and was vulnerable to confounding by indication. Patients with more active disease, greater chronic glucocorticoid exposure, previous infection, or more complex surgical requirements may have been more likely both to receive advanced therapy and to experience complications. Direct continuation-versus-withholding evidence was limited, and many studies evaluated medication exposure rather than a modifiable perioperative timing strategy. Disease duration could not be incorporated consistently into risk stratification because it was incompletely reported and was not a prespecified eligibility or subgroup variable.

The review retained supportive original studies concerning disease activity, surgical utilization, anatomical or procedure-related risk, and postoperative interpretation. These studies broadened the clinical context but were separated explicitly from direct medication evidence. In particular, the orthopedic surgery utilization study [42] and postoperative CRP kinetics study [41] were not used to formulate medication-timing recommendations or medication-effect certainty judgments.

Outcome definitions and follow-up periods varied substantially, particularly for SSI, PJI, delayed wound healing, and flare. Mixed inflammatory-arthritis cohorts introduced indirectness when RA-specific findings were not fully separable. Chronic baseline glucocorticoid exposure, short-term perioperative administration, and stress-dose supplementation were clinically distinct exposures; the review contained no direct comparison of stress-dose strategies.

Restriction to English-language full-text publications may have introduced language and availability bias. Study selection and data extraction were undertaken by one reviewer and checked by another rather than performed through fully independent duplicate review, creating a possibility of missed studies or extraction errors. The evidence-role classification should also be recognized as an interpretive synthesis method and does not overcome limitations in the underlying primary studies. Future prospective and randomized studies should use standardized outcome definitions and report exact medication withholding and restart intervals.

### 4.9. Future Directions

The next phase of research should move beyond broad retrospective comparisons and focus on prospective, surgery-specific, and drug-specific perioperative studies. Priority questions include whether one-dosing-interval withholding is sufficient across biologic classes, how to best manage JAK inhibitors and newer biologics, which procedures are most sensitive to flare-related functional decline, and how disease activity and glucocorticoid burden should be incorporated into perioperative risk stratification [16,18,30,54]. Standardized definitions of infection, delayed wound healing, and flare will be essential if future studies are to support credible meta-analysis and stronger guideline development. Until then, perioperative RA management will remain an area where the best decisions depend on integrating incomplete medication evidence with procedure-specific surgical judgment.

## 5. Conclusions

The certainty of evidence concerning perioperative antirheumatic medication management in adults with RA undergoing arthroplasty and non-arthroplasty orthopedic procedures remains very low. Methotrexate continuation was not associated with an evident increase in early complications, whereas direct evidence for other conventional synthetic DMARDs was limited. Longer interruption of biologic or targeted synthetic therapy did not consistently reduce infection or wound-healing complications and may increase postoperative flare. Chronic baseline glucocorticoid exposure was associated with adverse outcomes but should not be conflated with short-term perioperative administration; stress-dose supplementation was not directly evaluated. Medication decisions should therefore be individualized according to drug class, disease activity, infection history, and surgical procedure, with specific timing informed by procedure-specific guidance and multidisciplinary judgment.

## Figures and Tables

**Table 2:** GRADE assessment of certainty of evidence for the main outcomes.

Outcome	No. of Studies (Participants)	Direction of Evidence/Key Finding	Certainty of Evidence (GRADE)	Reasons for Rating
**Primary outcomes**				
Surgical site infection/periprosthetic joint infection	16 studies (participants not pooled because of heterogeneous and variably reported denominators)	No consistent evidence that prolonged interruption of modern antirheumatic therapy reduces postoperative infection risk; patient factors, steroid burden, revision status, and prior infection were often more predictive.	**Very low**	Started at low because the evidence base was predominantly observational. Downgraded for serious risk of bias due to retrospective non-randomized designs and confounding by indication; serious inconsistencies because procedure type, exposure definitions, and infection ascertainment varied; serious indirectness because some evidence came from procedure-specific or arthroplasty-dominant cohorts; and serious imprecision because event rates were low and estimates were unstable across studies. Publication bias could not be assessed formally.
Delayed wound healing	9 studies (participants/procedures not pooled)	Delayed wound healing appeared to be driven mainly by procedure type, anatomical site, operative burden, diabetes, and infection history; medication-specific effects were inconsistent.	**Very low**	Started at low. Downgraded for serious risk of bias; serious inconsistencies because wound-healing definitions varied markedly; serious indirectness because evidence came from mixed orthopedic and procedure-specific cohorts; and serious imprecision because many studies were small or event sparse. Publication bias could not be assessed formally.
Postoperative rheumatoid arthritis flare	5 studies (participants not pooled)	Interruption of biologic or targeted synthetic therapy may increase postoperative flare risk, especially with longer withholding intervals, but flare reporting was sparse and heterogeneous.	**Very low**	Started at low. Downgraded for serious risk of bias; serious inconsistencies because flare definitions and timing were not standardized; serious indirectness because several cohorts were procedure or drug-class specific; and serious imprecision because relatively few studies contributed direct flare data. Publication bias could not be assessed formally.
**Secondary outcomes**				
Reoperation/revision/readmission	7 studies (participants not pooled)	These outcomes appeared to be influenced more by implant mechanics, disease severity, revision context, and host factors than by a consistent perioperative medication effect.	**Very low**	Started at low. Downgraded for serious risk of bias, serious inconsistencies across procedure types and outcome definitions, serious indirectness because evidence was dominated by arthroplasty cohorts, and serious imprecision because no pooled estimate was possible and outcome events were variably reported.
Dislocation/mechanical complications	6 studies (participants not pooled)	Mechanical complications were most evident in hip and ankle arthroplasty and appeared to track more closely with disease activity, anatomy, and implant factors than with medication timing alone.	**Very low**	Started at low. Downgraded for serious risk of bias, serious indirectness because findings were procedure specific and not broadly generalizable, and serious imprecision because data were limited to a small number of cohorts with heterogeneous reporting.
Mortality/venous thromboembolism/other rare medical complications	2 original studies directly contributing outcome data, 1 guideline used for contextual interpretation only	Data were too sparse and inconsistent to support a reliable medication-specific conclusion for mortality or venous thromboembolism.	**Very low**	Started at low. Downgraded for serious risk of bias, serious indirectness, and very serious imprecision because events were infrequent and only a small number of studies contributed directly relevant outcome data. Publication bias could not be assessed formally. The contextual guideline was not counted as primary outcome evidence.

Table 2 Observational evidence was considered the dominant evidence base for all outcomes. Certainty was rated down for serious limitations in study design and execution, including retrospective non-randomized designs, confounding by indication, heterogeneous perioperative exposure definitions, variable procedure types, inconsistent outcome definitions, and imprecision arising from sparse event counts or limited direct comparative evidence. Supportive clinical-context studies were used to explain heterogeneity, potential confounding, and postoperative interpretation but were not used to determine the causal effect of medication continuation or withholding. Studies addressing orthopedic surgery utilization or postoperative biomarker kinetics did not contribute to medication-effect GRADE ratings.

**Table 3:** Clinically oriented summary of perioperative antirheumatic medication evidence in RA orthopedic surgery. Practical interpretations distinguish findings directly supported by included clinical studies from recommendations derived principally from guidelines or expert interpretation. Supportive clinical-context studies were not used as direct evidence for medication continuation or withholding.

Medication Class or Exposure	Evidence Base	Principal Surgical Settings	Strategy or Exposure Evaluated	Main Outcomes	Certainty	Practical Interpretation
**Methotrexate**	One randomized study with supportive observational evidence [3,22]	Mixed elective orthopedic surgery; hand surgery	Continuation versus withholding; continued perioperative therapy	No evident increase in early postoperative complications with continuation; withholding did not demonstrate a clear advantage	Very low overall; most direct evidence comes from one older trial	Direct evidence is most supportive of methotrexate continuation. Broader recommendations should still consider renal function, toxicity, and individual contraindications
**Other non-methotrexate csDMARDs**	Limited drug-specific observational evidence, principally leflunomide [23]	Arthroplasty and mixed orthopedic surgery	Continued exposure versus alternative or no exposure	No consistent infection increase identified, but the evidence base was sparse	Very low	Class-wide recommendations rely more heavily on guidelines than on comparative studies included in this review
**TNF inhibitors**	Multiple observational exposure, continuation, and timing studies [4,17,24,25,26,27,37,38,44]	THA/TKA, mixed orthopedic surgery, foot/ankle surgery, hand surgery	Continued versus unexposed treatment; shorter versus longer interruption	Infection findings were mixed; longer interruption did not consistently reduce SSI or PJI	Very low	The studies do not establish that prolonged interruption is safer. Exact withholding and restart intervals remain guideline derived
**Non-TNF biologics**	Abatacept timing study, tocilizumab cohorts, mixed biologic studies, and small ozoralizumab series [16,28,29,35,36,50,52]	Arthroplasty and mixed orthopedic surgery	Interruption timing, exposure, discontinuation, and restart	No consistent benefit from longer interruption; selected studies reported wound-healing concerns; flare occurred after interruption in some cohorts	Very low	Individualize according to drug, disease activity, infection history, and surgical setting. No universal interval is established
**JAK inhibitors**	Two small retrospective agent-specific studies and one broader contemporary cohort [18,30,31]	Mixed orthopedic surgery	Exposure, continuation/discontinuation, and interruption duration	Low absolute infection and wound-event rates; longer withholding associated with more flare in some analyses	Very low	Avoid assuming that longer interruption necessarily improves safety. Optimal timing remains uncertain
**Chronic baseline glucocorticoids**	Several observational dose and risk-factor studies [17,20,25,41,53]	Predominantly THA/TKA, mixed orthopedic surgery, and cervical spine surgery	Established daily exposure or dose strata	Higher baseline doses associated with infection, PJI, readmission, or other complications in several studies	Very low because of residual confounding, although the direction was relatively consistent	Chronic exposure is a potentially modifiable risk marker, but observational associations do not prove that rapid perioperative tapering improves outcomes
**Short-term perioperative glucocorticoids**	One retrospective arthroplasty study [32]	Total joint arthroplasty	Brief perioperative administration versus none	No increase in early complications or readmission; lower postoperative opioid requirements reported	Very low	Do not extrapolate risk estimates from chronic glucocorticoid therapy to a brief perioperative regimen
**Stress-dose glucocorticoid supplementation**	No direct eligible comparison	Not established	Stress-dose supplementation versus usual dose or no supplementation	Not directly evaluated	No direct evidence	No conclusion can be drawn from this review. Practice should be attributed to separate perioperative or endocrine guidance

## Data Availability

The data underlying this review are derived from published studies cited in the reference list. Study selection decisions, extracted study characteristics, risk-of-bias judgments, and search strategies are available from the corresponding author upon reasonable request.

## Supplementary Materials

The following supporting information can be downloaded at: https://www.mdpi.com/article/10.3390/medicina62081498/s1, Supplementary S1, complete electronic search strategies and pre-screening processing for PubMed, Scopus, and CENTRAL; Table S1, evidence-role classification of the 43 original clinical studies; Table S2, non-original contextual sources retained outside the original clinical-study evidence synthesis; PRISMA 2020 checklist.

## Abbreviations

**Abbreviation**	**Definition**
ACR	American College of Rheumatology
AAHKS	American Association of Hip and Knee Surgeons
RA	Rheumatoid arthritis
OA	Osteoarthritis
SLE	Systemic lupus erythematosus
DMARD	Disease-modifying antirheumatic drug
csDMARD	Conventional synthetic disease-modifying antirheumatic drug
bDMARD	Biologic disease-modifying antirheumatic drug
tsDMARD	Targeted synthetic disease-modifying antirheumatic drug
JAK	Janus kinase
JAKi	Janus kinase inhibitor
TNF	Tumor necrosis factor
TNFi	Tumor necrosis factor inhibitor
SSI	Surgical site infection
PJI	Periprosthetic joint infection
DWH	Delayed wound healing
VTE	Venous thromboembolism
DVT	Deep vein thrombosis
THA	Total hip arthroplasty
TKA	Total knee arthroplasty
TAA	Total ankle arthroplasty
TJA	Total joint arthroplasty
MCP	Metacarpophalangeal
SDAI	Simplified Disease Activity Index
CRP	C-reactive protein
WBC	White blood cell count
OR	Odds ratio
CI	Confidence interval
PRISMA	Preferred Reporting Items for Systematic Reviews and Meta-Analyses
PROSPERO	International Prospective Register of Systematic Reviews
GRADE	Grading of Recommendations Assessment, Development and Evaluation
NOS	Newcastle–Ottawa Scale
RoB 2	Cochrane Risk of Bias 2 tool
