# Supplementary material for: Perioperative Management of Antirheumatic Medications in Adults with Rheumatoid Arthritis Undergoing Arthroplasty and Other Orthopedic Procedures: A Systematic Review
Source: Medicina (Kaunas). 2026 Aug 4;62(8):1498. doi: 10.3390/medicina62081498 (PMC13514889; doi:10.3390/medicina62081498)
Supplement: Supplementary file 1 [file medicina-62-01498-s001.zip › medicina-4368685-supplementary.pdf]

Supplementary Materials

Perioperative Management of Antirheumatic Medications in Adults with Rheumatoid Arthritis Undergoing Arthroplasty and Other Orthopedic Procedures: A Systematic Review

Supplementary S1. Electronic Search Strategies and Pre-Screening Processing

A comprehensive literature search was conducted in PubMed, Scopus, and the Cochrane Central Register of Controlled Trials (CENTRAL) from database inception to 18 April 2026. The searches combined terms relating to rheumatoid arthritis, orthopedic surgery, perioperative management, and antirheumatic medication exposure. Eligibility was restricted to English-language full-text publications. Search syntax was adapted to the indexing structure and search functions of each database.

Before formal reviewer-based title and abstract screening, retrieved records were processed using prespecified database metadata, indexing terms, publication-type filters, and keyword-based scope rules. Records were removed when these criteria identified non-English-language publications, ineligible publication types, non-human or laboratory-only studies, or records clearly outside the predefined rheumatoid arthritis–orthopedic surgery scope. These pre-screening categories collectively accounted for 479 records. No machine-learning or automated eligibility-classification tools were used for formal title and abstract screening or full-text eligibility assessment.

PubMed search strategy:

(("Arthritis, Rheumatoid"[Mesh] OR "rheumatoid arthritis"[tiab])

AND

("Arthroplasty, Replacement, Hip"[Mesh] OR "Arthroplasty, Replacement, Knee"[Mesh] OR arthroplasty[tiab] OR "total hip arthroplasty"[tiab] OR "total knee arthroplasty"[tiab] OR "total ankle arthroplasty"[tiab] OR "foot surgery"[tiab] OR "ankle surgery"[tiab] OR "hand surgery"[tiab] OR "wrist surgery"[tiab] OR "cervical spine surgery"[tiab] OR "spine surgery"[tiab] OR "orthopedic surgery"[tiab] OR "orthopaedic surgery"[tiab])

AND

(perioperat*[tiab] OR preoperat*[tiab] OR postoperat*[tiab] OR "drug interruption"[tiab] OR "drug discontinuation"[tiab] OR "drug continuation"[tiab] OR "medication withholding"[tiab] OR "perioperative management"[tiab])

AND

(methotrexate[tiab] OR leflunomide[tiab] OR biologic*[tiab] OR infliximab[tiab] OR etanercept[tiab] OR adalimumab[tiab] OR abatacept[tiab] OR tocilizumab[tiab] OR rituximab[tiab] OR "JAK inhibitor*"[tiab]

OR tofacitinib[tiab] OR baricitinib[tiab] OR upadacitinib[tiab] OR glucocorticoid*[tiab] OR steroid*[tiab] OR ozoralizumab[tiab]))

Scopus search strategy:

TITLE-ABS-KEY ("rheumatoid arthritis")

AND TITLE-ABS-KEY (arthroplasty OR "total hip arthroplasty" OR "total knee arthroplasty" OR "foot surgery" OR "ankle surgery" OR "hand surgery" OR "orthopedic surgery")

AND TITLE-ABS-KEY (perioperat* OR preoperat* OR postoperat*)

AND TITLE-ABS-KEY (methotrexate OR biologic* OR infliximab OR abatacept OR tocilizumab OR "JAK inhibitor*" OR glucocorticoid* OR steroid*)

CENTRAL search strategy:

("rheumatoid arthritis")

AND

(arthroplasty OR "total hip arthroplasty" OR "total knee arthroplasty" OR "total ankle arthroplasty" OR "foot surgery" OR "ankle surgery" OR "hand surgery" OR "wrist surgery" OR "spine surgery" OR "orthopedic surgery" OR "orthopaedic surgery")

AND

(perioperative OR preoperative OR postoperative OR "drug interruption" OR "drug discontinuation" OR "drug continuation" OR "perioperative management")

AND

(methotrexate OR leflunomide OR biologic* OR infliximab OR etanercept OR adalimumab OR abatacept OR tocilizumab OR rituximab OR tofacitinib OR baricitinib OR upadacitinib OR glucocorticoid* OR steroid*).

Supplementary Table S1. Evidence-role classification of the 43 original clinical studies

Reference	Study	Main exposure or contextual factor	Evidence role	Contribution to the synthesis	Medication-effect GRADE
Ref 3	Barnard et al., 2012	Perioperative continuation of antirheumatic medications except injectable biologics	D	Direct perioperative strategy evidence concerning wound healing	Yes, outcome-dependent
Ref 4	Bibbo et al., 2004	TNF-inhibitor exposure during foot and ankle surgery	E	Medication-exposure evidence concerning infection and healing	Yes, outcome-dependent

Reference	Study	Main exposure or contextual factor	Evidence role	Contribution to the synthesis	Medication-effect GRADE
Ref 5	Hirao et al., 2017	Biologic-era outcomes after total ankle arthroplasty	S	Procedure-specific arthroplasty context	No
Ref 6	Cathey et al., 2026	Perioperative biologic exposure in elective hand surgery	E	Medication-exposure evidence concerning infection and wound complications	Yes, outcome-dependent
Ref 7	Klifto et al., 2020	Perioperative immunosuppressant exposure in hand surgery	E	Medication-exposure evidence concerning postoperative complications	Yes, outcome-dependent
Ref 16	Nishida et al., 2026	Ozoralizumab discontinuation and postoperative restart	D	Direct perioperative strategy evidence concerning SSI, DWH, and flare	Yes, outcome-dependent
Ref 17	Gilson et al., 2010	TNF-blocker exposure and chronic glucocorticoid burden	E	Medication-exposure and dose-associated infection evidence	Yes, outcome-dependent
Ref 18	Ito et al., 2025	Continuation versus discontinuation of bDMARDs/tsDMARDs	D	Direct strategy evidence concerning SSI, DWH, mortality, and flare	Yes, outcome-dependent
Ref 19	Lai YT et al., 2024	Preoperative and postoperative RA disease activity	S	Disease-activity and complication-risk context	No
Ref 20	George et al., 2019	Biologic-agent exposure and chronic glucocorticoid dose	E	Medication-exposure and glucocorticoid dose–response evidence	Yes, outcome-dependent
Ref 21	Mori et al., 2025	RA versus osteoarthritis after total hip arthroplasty	S	Disease-cohort and procedure-specific complication context	No
Ref 22	Grennan et al., 2001	Methotrexate continuation versus withholding	D	Direct randomized strategy evidence	Yes

Reference	Study	Main exposure or contextual factor	Evidence role	Contribution to the synthesis	Medication-effect GRADE
				concerning complications and flare	
Ref 23	Tanaka et al., 2003	Continued leflunomide exposure	D	Direct comparative evidence concerning infectious complications	Yes, outcome-dependent
Ref 24	den Broeder et al., 2007	Perioperative anti-TNF continuation	D	Direct strategy evidence concerning SSI and other complications	Yes, outcome-dependent
Ref 25	George et al., 2017	Shorter versus longer infliximab interruption	D	Direct timing evidence concerning serious infection and PJI	Yes, outcome-dependent
Ref 26	Hayata et al., 2011	Timing of surgery after infliximab treatment	D	Direct interval evidence concerning perioperative outcomes	Yes, outcome-dependent
Ref 27	Kawakami et al., 2010	Perioperative interruption of TNF blockers	D	Direct strategy evidence concerning SSI and complications	Yes, outcome-dependent
Ref 28	George et al., 2019	Shorter versus longer abatacept interruption	D	Direct timing evidence concerning postoperative infection and other outcomes	Yes, outcome-dependent
Ref 29	Momohara et al., 2013	Tocilizumab exposure	E	Medication-exposure evidence concerning perioperative clinical features and complications	Yes, outcome-dependent
Ref 30	Bekki et al., 2024	JAK-inhibitor versus bDMARD exposure	E	Medication-exposure evidence concerning complications and flare	Yes, outcome-dependent

Reference	Study	Main exposure or contextual factor	Evidence role	Contribution to the synthesis	Medication-effect GRADE
Ref 31	Nishida et al., 2024	JAK-inhibitor interruption duration	D	Direct interval evidence concerning complications and flare	Yes, outcome-dependent
Ref 32	Lai YH et al., 2024	Short-term perioperative glucocorticoid administration	D	Direct perioperative administration evidence concerning early complications and readmission	Yes, outcome-dependent
Ref 33	Yi et al., 2025	Medication modification versus no modification	D	Direct strategy evidence concerning early complications and DWH	Yes, outcome-dependent
Ref 34	Kadota et al., 2016	Multiple medication, patient, and operative risk factors	S	Supportive risk-modifier evidence concerning SSI and DWH	No
Ref 35	Kiso et al., 2025	bDMARD-exposed versus unexposed procedures	E	Medication-exposure evidence concerning SSI and DWH	Yes, outcome-dependent
Ref 36	Kubota et al., 2012	Biologic versus non-biologic exposure	E	Medication-exposure evidence concerning DWH and infection	Yes, outcome-dependent
Ref 37	Momohara et al., 2011	Biologic versus non-biologic DMARD exposure	E	Medication-exposure evidence concerning acute SSI and PJI	Yes, outcome-dependent
Ref 38	Suzuki et al., 2011	Biologic versus non-biologic treatment exposure	E	Medication-exposure evidence concerning infection and postoperative complications	Yes, outcome-dependent
Ref 39	Tada et al., 2016	Biologic exposure and other perioperative factors	E	Medication-exposure evidence with additional risk-factor analyses	Yes, outcome-dependent

Reference	Study	Main exposure or contextual factor	Evidence role	Contribution to the synthesis	Medication-effect GRADE
Ref 40	Yano et al., 2016	Operative and patient factors in forefoot surgery	S	Procedure-specific DWH risk-modifier evidence	No
Ref 41	Kishimoto et al., 2022	Postoperative CRP kinetics, BMI, and glucocorticoid exposure	S	Postoperative biomarker-interpretation context	No
Ref 42	Richter et al., 2018	Demographic and disease-related determinants of orthopedic surgery utilization	S	Surgical-utilization, disease-burden, and patient-selection context	No
Ref 43	Bongartz et al., 2008	Clinical risk factors for PJI after THA/TKA	S	Previous infection, revision, and arthroplasty-risk context	No
Ref 44	Borgas et al., 2020	Antirheumatic treatment exposure, including TNF inhibitors	E	Medication-exposure evidence concerning PJI	Yes, outcome-dependent
Ref 45	Chung et al., 2021	RA versus osteoarthritis after total knee arthroplasty	S	Disease-cohort and baseline surgical-risk context	No
Ref 46	Cordtz et al., 2018	Preoperative treatment exposure, including biologics	E	Medication-exposure evidence concerning revision, PJI, and mortality	Yes, outcome-dependent
Ref 47	Cordtz et al., 2020	RA treatment-era complications after THA/TKA	S	Disease-cohort and medical-complication context	No
Ref 48	Di Martino et al., 2023	Perioperative TNF-inhibitor exposure in total hip arthroplasty	E	Medication-exposure evidence concerning infection, revision, and survival	Yes, outcome-dependent
Ref 49	Takakubo et al., 2024	Biologic-era THA and revision outcomes	S	Arthroplasty and revision-risk context	No

Reference	Study	Main exposure or contextual factor	Evidence role	Contribution to the synthesis	Medication-effect GRADE
Ref 50	Kubota et al., 2014	Biologic versus non-biologic exposure	E	Medication-exposure evidence concerning postoperative infection	Yes, outcome-dependent
Ref 51	Yamada et al., 2020	Preoperative biologic/tsDMARD exposure, surgery type, and postoperative disease activity	S	Disease activity and postoperative clinical-context evidence	No
Ref 52	Okita et al., 2021	Biologic exposure, including tocilizumab	E	Medication-exposure evidence concerning DWH	Yes, outcome-dependent
Ref 53	Sakuraba et al., 2022	Glucocorticoid burden and other cervical-spine risk factors	S	Procedure-specific complication-risk context	No

Evidence-role definitions: D, direct perioperative strategy evidence evaluating medication continuation, withholding, interruption duration, restart timing, medication modification, or short-term perioperative administration; E, medication-exposure evidence comparing medication-exposed and unexposed groups, medication classes, or dose strata; S, supportive clinical-context evidence concerning disease status, surgical utilization, patient or procedural risk, operative complexity, or postoperative interpretation. “Yes, outcome-dependent” indicates that the study was eligible to contribute to an outcome-level medication-effect GRADE assessment only when it reported the relevant postoperative clinical outcome.

Supplementary Table S2. Non-original contextual sources retained outside the original clinical-study evidence synthesis

Reference	Evidence type	Role in the manuscript
Ref 8	Focused review	Contextual interpretation of perioperative antirheumatic medication management in foot and ankle surgery
Ref 11	2017 ACR/AAHKS clinical practice guideline	Perioperative medication recommendation framework for elective THA and TKA
Ref 12	2022 ACR/AAHKS clinical practice guideline update	Updated perioperative medication recommendation framework for elective THA and TKA

Reference	Evidence type	Role in the manuscript
Ref 13	Systematic evidence review and guideline-support source	Contextual risk-factor and evidence synthesis supporting the Japan College of Rheumatology recommendations
Ref 14	Systematic review/meta-analytic evidence source	Contextual evidence concerning perioperative anti-TNF exposure, treatment interruption, and postoperative infection
Ref 54	Randomized trial protocol	Ongoing direct evidence concerning perioperative continuation versus withholding of biologic therapy

These six sources were retained only for guideline interpretation, comparison with previous evidence syntheses, and identification of research gaps. They were not included among the 43 original clinical studies and did not contribute to the risk-of-bias assessment or medication-effect GRADE ratings.
