# Supplementary material for: Perioperative Management of Antirheumatic Medications in Adults with Rheumatoid Arthritis Undergoing Arthroplasty and Other Orthopedic Procedures: A Systematic Review
Source: Medicina (Kaunas). 2026 Aug 4;62(8):1498. doi: 10.3390/medicina62081498 (PMC13514889; doi:10.3390/medicina62081498)
Supplement: Supplementary file 1 [file medicina-62-01498-s001.zip › PRISMA_2020_checklist.pdf]

PRISMA 2020 Checklist

Manuscript: Perioperative Management of Antirheumatic Medications in Rheumatoid Arthritis Patients Undergoing Orthopedic Surgery: A Systematic Review.

Section and Topic	Item #	Checklist item	
**TITLE**	**TITLE**	**TITLE**	
Title	1	Identify the report as a systematic review.	Manuscript p. 1 (title page): the title identifies the article as a “Systematic Review”.
**ABSTRACT**	**ABSTRACT**	**ABSTRACT**	
Abstract	2	See the PRISMA 2020 for Abstracts checklist.	Manuscript p. 1 (Abstract): reports the background/rationale, eligibility scope, outcomes, number of included studies/contextual sources, main findings, and conclusion.
**INTRODUCTION**	**INTRODUCTION**	**INTRODUCTION**	
Rationale	3	Describe the rationale for the review in the context of existing knowledge.	Manuscript pp. 1-3 (Introduction, paragraphs 1-6): explains the RA orthopedic burden, perioperative medication dilemma, guideline limitations, fragmented evidence, and need for an RA-focused orthopedic synthesis.
Objectives	4	Provide an explicit statement of the objective(s) or question(s) the review addresses.	Manuscript p. 3 (Introduction, final paragraph): states the objective of evaluating perioperative antirheumatic medication management in adults with RA undergoing orthopedic surgery, including primary and secondary outcomes.
**METHODS**	**METHODS**	**METHODS**	
Eligibility criteria	5	Specify the inclusion and exclusion criteria for the review and how studies were grouped for the syntheses.	Manuscript pp. 3-6 (Methods Sections 2.2 PICOS Statement, 2.3 Eligibility Criteria, and 2.8 Data Synthesis and Statistical Analysis): gives inclusion/exclusion criteria and explains grouping by outcome domain, medication class, procedure type, and continuation/withholding strategy.
Information sources	6	Specify all databases, registers, websites, organisations, reference lists and other sources searched or consulted to identify studies. Specify the date when each source was last searched or consulted.	Manuscript p. 4 (Methods Section 2.4 Information Sources and Search Strategy): reports PubMed, Scopus, CENTRAL, database inception to 18 April 2026, English full-text limit, manual reference-list screening, and forward citation tracking.
Search strategy	7	Present the full search strategies for all databases, registers and websites, including any filters and limits used.	Manuscript p. 4 (Methods Section 2.4) and Supplementary Appendix S1: the manuscript states that full PubMed, Scopus, and CENTRAL strategies are provided in Appendix S1, including exact search strings, filters, and limits.
Selection process	8	Specify the methods used to decide whether a study met the inclusion criteria of the review, including how many reviewers screened each record and each report retrieved, whether they worked independently, and if applicable, details of automation tools used in the process.	Manuscript pp. 4-5 (Methods Section 2.5 Study Selection): describes two-stage title/abstract and full-text screening, duplicate removal, one reviewer with checking by at least one additional reviewer, uncertainty resolution by discussion, avoidance of duplicate outcome datasets, and no automation tools were used.
Data collection process	9	Specify the methods used to collect data from reports, including how many reviewers collected data from each report, whether they worked independently, any processes for obtaining or confirming data from study investigators, and if applicable, details of automation tools used in the process.	Manuscript p. 5 (Methods Section 2.6 Data Extraction) and p. 5 (Section 2.7 Risk of Bias Assessment): describes standardized extraction, one reviewer with checking by at least one additional reviewer, variables extracted, individual participant data not sought, no additional investigator information sought, no automation tools were used.
Data items	10a	List and define all outcomes for which data were sought. Specify whether all results that were compatible with each outcome domain in each study were sought (e.g. for all measures, time points, analyses), and if not, the methods used to decide which results to collect.	Manuscript pp. 3, 5-6 (Methods Sections 2.2, 2.6, and 2.8): defines primary outcomes (SSI/PJI, DWH, RA flare), secondary outcomes, compatible postoperative results across definitions/time points/analyses, and prioritization of adjusted estimates when available.
Data items	10b	List and define all other variables for which data were sought (e.g. participant and intervention characteristics, funding sources). Describe any assumptions made about any missing or unclear information.	Manuscript p. 5 (Methods Section 2.6 Data Extraction): lists study characteristics, clinical setting, medication exposure, comparator, follow-up, quantitative data, disease activity, glucocorticoid burden, comorbidities, operative time, revision status, and outcome definitions.

			Added wording states that missing or unclear information was recorded as not reported and no assumptions/imputations were made.
Study risk of bias assessment	11	Specify the methods used to assess risk of bias in the included studies, including details of the tool(s) used, how many reviewers assessed each study and whether they worked independently, and if applicable, details of automation tools used in the process.	Manuscript p. 5 (Methods Section 2.7 Risk of Bias Assessment): reports RoB 2 for randomized studies, Newcastle-Ottawa Scale for non-randomized cohort/case-control studies, two reviewers with checking by at least one additional reviewer, assessed bias domains, and the added statement that no automation tools were used.
Effect measures	12	Specify for each outcome the effect measure(s) (e.g. risk ratio, mean difference) used in the synthesis or presentation of results.	Manuscript p. 6 (Methods Section 2.8 Data Synthesis and Statistical Analysis): reports odds ratios, hazard ratios, risk ratios, proportions, and raw event counts as the effect measures extracted/presented depending on study design and reporting.
Synthesis methods	13a	Describe the processes used to decide which studies were eligible for each synthesis (e.g. tabulating the study intervention characteristics and comparing against the planned groups for each synthesis (item #5)).	Manuscript pp. 4-6 (Methods Sections 2.3 and 2.8): explains that synthesis eligibility was determined by original clinical study status, RA orthopedic population, medication exposure, postoperative outcomes, and grouping by outcome, medication class, procedure type, and strategy.
Synthesis methods	13b	Describe any methods required to prepare the data for presentation or synthesis, such as handling of missing summary statistics, or data conversions.	Manuscript pp. 5-6 (Methods Sections 2.6 and 2.8): adjusted measures were prioritized over crude data and quantitative pooling was not performed because of heterogeneity. Added wording states that no imputation, data conversion, or transformation was performed and missing summary statistics were reported as unavailable.
Synthesis methods	13c	Describe any methods used to tabulate or visually display results of individual studies and syntheses.	Manuscript pp. 6-25: methods for display are in Section 2.8 (p. 6); Figure 1 PRISMA flow diagram and Section 3.1 are on p. 7; Table 1A/1B study characteristics are on pp. 7-15; Figure 2 risk-of-bias visualization is on pp. 15-17; Table 2 GRADE summary is on pp. 23-25.
Synthesis methods	13d	Describe any methods used to synthesize results and provide a rationale for the choice(s). If meta-analysis was performed, describe the model(s), method(s) to identify the presence and extent of statistical heterogeneity, and software package(s) used.	Manuscript p. 6 (Methods Section 2.8): states that structured narrative synthesis was used because of substantial clinical and methodological heterogeneity in procedure type, medication class, comparator strategy, and outcome definitions; no meta-analysis was performed because sufficient homogeneity was not identified.
Synthesis methods	13e	Describe any methods used to explore possible causes of heterogeneity among study results (e.g. subgroup analysis, meta-regression).	Manuscript pp. 6 and 21-23 (Methods Section 2.8 and Results Sections 3.6-3.7): heterogeneity was explored descriptively by medication class, procedure type, continuation/discontinuation strategy, outcome domain, disease activity, steroid burden, revision status, and operative complexity.
Synthesis methods	13f	Describe any sensitivity analyses conducted to assess robustness of the synthesized results.	Manuscript p. 6 (Methods Section 2.8): added wording states that no formal sensitivity analyses were performed because no meta-analysis or pooled statistical synthesis was conducted.
Reporting bias assessment	14	Describe any methods used to assess risk of bias due to missing results in a synthesis (arising from reporting biases).	Manuscript pp. 5 and 23 (Methods Section 2.7 and Results Section 3.8 Reporting Bias Assessment): missing results were considered through selective/incomplete outcome reporting; formal outcome-level reporting-bias assessment was limited because meta-analysis was not performed.
Certainty assessment	15	Describe any methods used to assess certainty (or confidence) in the body of evidence for an outcome.	Manuscript p. 6 (Methods Section 2.8): describes GRADE certainty assessment using risk of bias, inconsistency, indirectness, imprecision, and suspected reporting/publication bias.
**RESULTS**	**RESULTS**	**RESULTS**	
Study selection	16a	Describe the results of the search and selection process, from the number of records identified in the search to the number of studies included in the review, ideally using a flow diagram.	Manuscript p. 7 (Figure 1 PRISMA flow diagram and Results Section 3.1 Study Selection): reports 796 records identified, 211 duplicates removed, 479 removed for other reasons, 106 screened, 57 excluded, 49 full texts retrieved/assessed, 43 original studies included, and 6 contextual sources retained.
Study selection	16b	Cite studies that might appear to meet the inclusion criteria, but which were excluded, and explain why they were excluded.	Manuscript p. 7 (Results Section 3.1 and Figure 1): excluded/retained contextual reports are reported by reason: protocol (n=1), guidelines (n=2), systematic review/meta-analysis sources (n=2), and focused review (n=1). these contextual sources are [49], [11,12], [9,10], and [8].

Study characteristics	17	Cite each included study and present its characteristics.	Manuscript pp. 7-15 (Results Section 3.2 Study Characteristics and Table 1A/1B): each included study is cited and summarized by author/year, surgery type, study design, population, sample size, exposure strategy, comparator, and outcomes.
Risk of bias in studies	18	Present assessments of risk of bias for each included study.	Manuscript pp. 15-17 (Results Section 3.3 Quality Assessment and Figure 2): presents risk-of-bias judgments for the original clinical studies, including RoB 2 for the randomized methotrexate study and NOS-based traffic-light judgments for non-randomized studies.
Results of individual studies	19	For all outcomes, present, for each study: (a) summary statistics for each group (where appropriate) and (b) an effect estimate and its precision (e.g. confidence/credible interval), ideally using structured tables or plots.	Manuscript pp. 17-25 (Results Sections 3.4-3.7 and Table 2), with supporting study characteristics on pp. 7-15: reports individual study findings, event counts, proportions, odds ratios, confidence intervals, p values, and qualitative results where available.
Results of syntheses	20a	For each synthesis, briefly summarise the characteristics and risk of bias among contributing studies.	Manuscript pp. 17-25 (Results Sections 3.4-3.7 and Table 2): summarizes contributing study types, procedure/medication context, methodological limitations, consistency of findings, and risk-of-bias implications for each synthesis domain.
Results of syntheses	20b	Present results of all statistical syntheses conducted. If meta-analysis was done, present for each the summary estimate and its precision (e.g. confidence/credible interval) and measures of statistical heterogeneity. If comparing groups, describe the direction of the effect.	Manuscript pp. 17-25 (Results Sections 3.4-3.7 and Table 2): no meta-analysis or pooled statistical synthesis was conducted; narrative synthesis results and direction of effect are presented for each outcome domain.
Results of syntheses	20c	Present results of all investigations of possible causes of heterogeneity among study results.	Manuscript pp. 21-23 (Results Sections 3.6 and 3.7): presents descriptive exploration of heterogeneity by medication class, surgery type, disease activity, glucocorticoid burden, revision/recent arthroplasty status, and operative complexity.
Results of syntheses	20d	Present results of all sensitivity analyses conducted to assess the robustness of the synthesized results.	Manuscript pp. 6 and 23 (Methods Section 2.8 and Results Section 3.7): no formal sensitivity analyses were performed because the review did not conduct meta-analysis or pooled statistical synthesis.
Reporting biases	21	Present assessments of risk of bias due to missing results (arising from reporting biases) for each synthesis assessed.	Manuscript p. 23 (Results Section 3.8 Reporting Bias Assessment): reports that formal assessment was limited because no meta-analysis was performed and notes incomplete outcome reporting and emphasis on infection over wound-healing/flare outcomes.
Certainty of evidence	22	Present assessments of certainty (or confidence) in the body of evidence for each outcome assessed.	Manuscript pp. 23-25 (Results Section 3.7 and Table 2): presents GRADE certainty for SSI/PJI, DWH, RA flare, reoperation/revision/readmission, mechanical complications, and mortality/VTE/rare medical complications.
**DISCUSSION**	**DISCUSSION**	**DISCUSSION**	
Discussion	23a	Provide a general interpretation of the results in the context of other evidence.	Manuscript pp. 25-29 (Discussion Sections 4.1-4.5): interprets findings in relation to csDMARD continuation, biologic/tsDMARD interruption, infection-wound-flare tradeoff, host/surgical factors, and guideline scope.
Discussion	23b	Discuss any limitations of the evidence included in the review.	Manuscript p. 29 (Discussion Section 4.8 Strengths and Limitations): discusses limitations of the included evidence, including retrospective designs, confounding by indication, heterogeneous outcome definitions, variable follow-up, and possible overlapping clinical ecosystems.
Discussion	23c	Discuss any limitations of the review processes used.	Manuscript p. 29 (Discussion Section 4.8 Strengths and Limitations): discusses review-process limitations, including single-reviewer screening/data extraction checked by an additional reviewer and the risk of missed records or extraction error.
Discussion	23d	Discuss implications of the results for practice, policy, and future research.	Manuscript pp. 28-30 (Discussion Section 4.6 Clinical Implications, Section 4.9 Future Directions, and Conclusion): discusses implications for individualized orthopedic-rheumatology planning, glucocorticoid minimization, and future prospective standardized studies.
**OTHER INFORMATION**	**OTHER INFORMATION**	**OTHER INFORMATION**	
Registration and protocol	24a	Provide registration information for the review, including register name and registration number, or state that the review was not registered.	Manuscript p. 3 (Methods Section 2.1 Study Design and Registration): reports PROSPERO registration number CRD420261372707.

Registration and protocol	24b	Indicate where the review protocol can be accessed, or state that a protocol was not prepared.	Manuscript p. 3 (Methods Section 2.1 Study Design and Registration): “The protocol is available through the PROSPERO record (CRD420261372707).”
Registration and protocol	24c	Describe and explain any amendments to information provided at registration or in the protocol.	Manuscript p. 3 (Methods Section 2.1 Study Design and Registration): “No amendments were made after registration.”
Support	25	Describe sources of financial or non-financial support for the review, and the role of the funders or sponsors in the review.	Manuscript pp. 30-31 (Funding section): reports “No specific funding was received for this systematic review.”
Competing interests	26	Declare any competing interests of review authors.	Manuscript pp. 30-31 (Conflicts of Interest section): declares that the authors have no competing interests.
Availability of data, code and other materials	27	Report which of the following are publicly available and where they can be found: template data collection forms; data extracted from included studies; data used for all analyses; analytic code; any other materials used in the review.	Manuscript pp. 30-31 (Data Availability Statement): states that data are derived from published studies and that study-selection decisions, extracted characteristics, risk-of-bias judgments, and search strategies are available on request.

Checklist source: PRISMA 2020 checklist adapted from Page MJ, McKenzie JE, Bossuyt PM, Boutron I, Hoffmann TC, Mulrow CD, et al. BMJ 2021;372:n71. doi: 10.1136/bmj.n71. Licensed under CC BY 4.0.
